# A monomeric envelope glycoprotein cytoplasmic tail is sufficient for HIV-1 Gag lattice trapping and incorporation

**DOI:** 10.1128/jvi.02105-24

**Published:** 2025-04-15

**Authors:** Nicholas S. Groves, Austin R. Clark, Rebekah S. Aguilar, Yuta Hikichi, Anastasiia Kostenko, Merissa M. Bruns, Alegra T. Aron, Eric O. Freed, Schuyler B. van Engelenburg

**Affiliations:** 1 Molecular and Cellular Biophysics Program, Department of Biological Sciences, University of Denver 2927, Denver, Colorado, USA; 2 Virus-Cell Interaction Section, HIV Dynamics and Replication Program, Center for Cancer Research, National Cancer Institute 70717, Frederick, Maryland, USA; 3 Department of Chemistry and Biochemistry, University of Denver 2927, Denver, Colorado, USA; Icahn School of Medicine at Mount Sinai, New York, New York, USA

**Keywords:** HIV-1, Gag, Env, single-particle tracking, biochemistry, virus assembly, glycoprotein incorporation

## Abstract

To become infectious, assembling enveloped viruses must acquire viral glycoproteins to mediate downstream infection events. Human immunodeficiency virus-1 (HIV-1) envelope glycoproteins (Env) are well characterized to function as trimers for membrane fusion and entry; however, we sought to understand whether the trimeric structure of Env is required for incorporation into virus particles. Using superresolution live-cell imaging and biochemical assays, we demonstrate that a monomeric receptor chimera containing the Env cytoplasmic tail (Env-CT), known to regulate Env incorporation, is sufficient for lattice trapping and incorporation into virus assembly sites. We also demonstrate that these Env-CT monomers can restrict the incorporation of native Env trimers, competing for an apparently limited number of interaction sites in each assembling particle. Furthermore, this monomeric construct can restrict the incorporation of Env glycoproteins from an evolutionarily distant HIV-1 primary isolate. Our findings support a model where a monomeric Env-CT mediates Env incorporation, with this mechanism of Env incorporation being conserved between distant clades of HIV-1.

**IMPORTANCE:**

To combat the prevalence of HIV-1 and antiviral resistance, new classes of antivirals are needed. An attractive target for new classes includes virus assembly because released virus particles unable to obtain Env glycoproteins are non-infectious and unable to propagate HIV-1 infection. One requisite to the development of an antiviral targeting Gag-Env coalescence is the need to define the functional units constituting this molecular interface. Although Env functions as an obligatory trimer for virus entry, we demonstrate that a monomeric Env-CT is sufficient for Env incorporation into HIV-1 particles. Monomeric Env-CT displayed saturability in viral lattices and the ability to compete with native Env trimers for particle incorporation. These results suggest a less complex Env-CT structure mediates virus incorporation and that Env-CT mimetics could yield broad competitive activity against HIV-1 infection.

## INTRODUCTION

During HIV-1 infection, the trimeric viral envelope glycoprotein, Env, and the viral core protein, Gag, are biosynthesized and trafficked to the plasma membrane through independent pathways. Upon reaching the plasma membrane, Gag assembles into a lattice that deforms the membrane into a bud with outward curvature (1, 2). Env is co-translationally folded in the endoplasmic reticulum and is cleaved by host Furin proteases into the soluble gp120 and membrane-anchored gp41 fragments (3, 4). The functional oligomerized gp120/gp41 heterodimeric trimer then traffics through the secretory pathway to the plasma membrane where it then laterally diffuses on the surface until encountering a Gag lattice (1, 5–10). We have previously observed the trapping of native Env trimers at HIV-1 assembly sites and their confinement to a sub-viral region on the virus bud (11, 12), indicating that Env is retained in the Gag lattice through either a direct interaction or lattice corralling, as has been suggested previously (8, 13–15). We observed that this lattice confinement is dependent on the Env-CT and Gag matrix (MA) domains (11); however, the conformational and oligomeric states required for lattice trapping of Env remain unknown.

The HIV-1 Env ectodomain functions as a trimer, with sequences in the gp41 ectodomain and transmembrane domain (TMD) driving oligomerization to mediate virus membrane fusion with a naïve target cell (16–20). It has also been shown that the Env-CT can influence the ectodomain conformation and fusogenicity of Env, suggesting that the structure of the Env-CT may act as a molecular switch to regulate Env function (18, 21). The first *in vitro* solution structure of the Env-CT, in the absence of the transmembrane domain, depicts a relatively linear conformation with amphipathic α-helical secondary structure embedded in a liposomal membrane (22). A subsequent additional solution structure of the Env TMD and the CT demonstrated that the Env-CT, when trimerized by the TMD, can form a spiral-like ternary complex, termed a “baseplate,” juxtaposed with the inner leaflet of the membrane (23). This latter study demonstrated that perturbations to the quaternary baseplate contact sites can influence ectodomain conformations. Collectively, these observations could suggest that the Env-CT may undergo conformational changes between linear and quaternary structures at different stages of the infection cycle. Herein, we address whether the quaternary structure of the Env-CT is necessary for Env incorporation into assembling HIV-1 virions.

## RESULTS

### Validation of monomeric HIV-1 Env-CT chimeras for EnvCT-dependent virus incorporation

**Fig 1:**
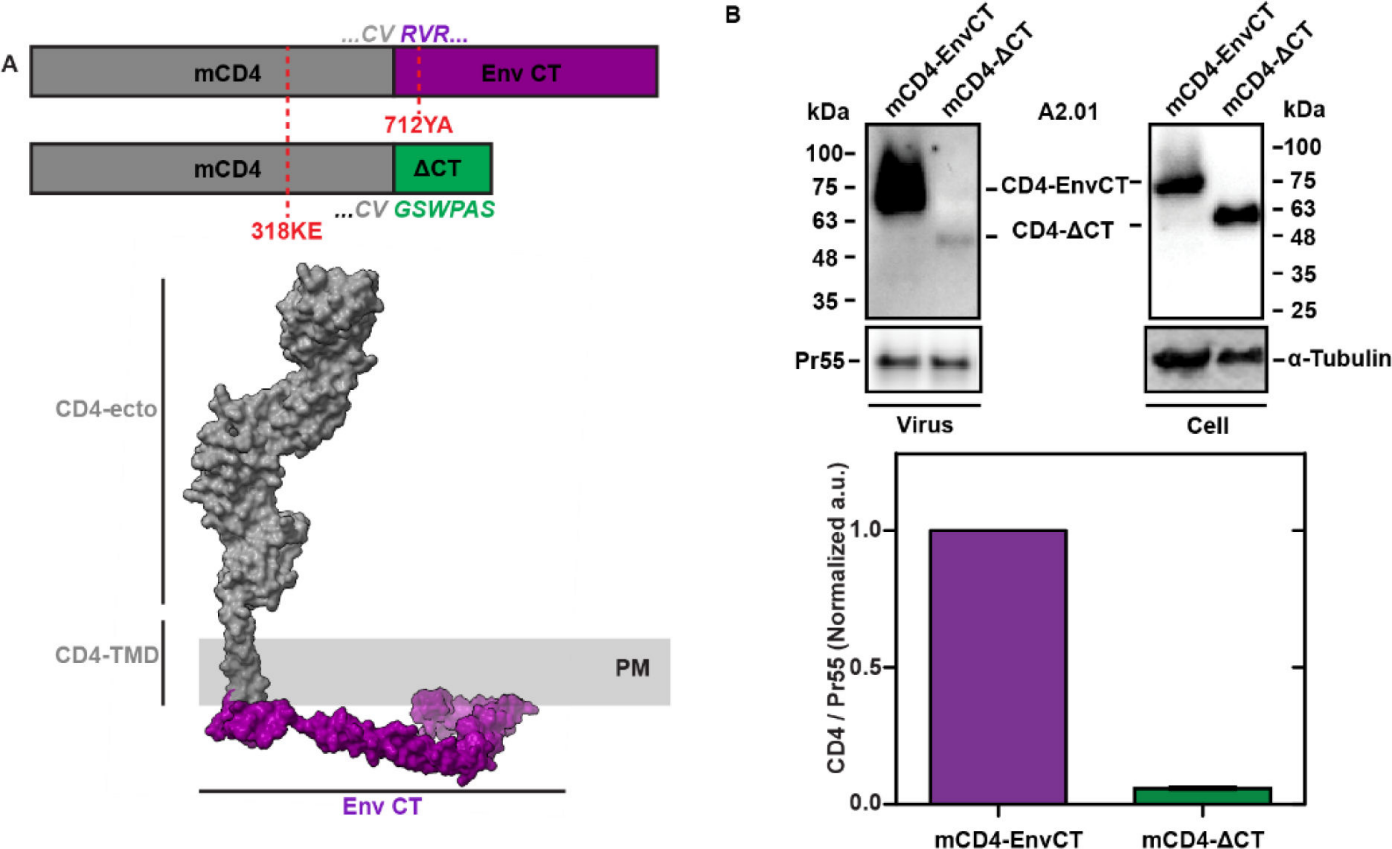
Monomeric HIV-1 Env cytoplasmic tail chimeras are incorporated into virus particles in an Env-CT-dependent manner. (A) Genetic design of a monomeric CD4-EnvCT 712YA chimera and hypothetical domain organization (Env-CT: PDBID: 5VWL, CD4: PDBID:1WIP). The GSWPAS peptide motif is the epitope for the anti-EnvCT antibody Chessie8 and is appended to the mCD4-∆CT construct to enable comparative Western blotting. (B) A2.01 T-cell lines stably expressing mCD4-EnvCT or mCD4-∆CT were transduced with NL4-3 virus (∆*pol*/∆*vif*/∆*vpr*/∆*env*). After 48 hours, virus and cell samples were analyzed by Western blot using anti-CD4, anti-p24, and anti-tubulin antibodies (top). The A2.01 T-cell line is null for native CD4. HIV-1 particle incorporation of mCD4-EnvCT in the A2.01 T-cell line is strongly dependent on the Env-CT, suggesting that a monomeric Env-CT is sufficient for Gag-lattice enrichment (mCD4-EnvCT /mCD4-∆CT = 16.7-fold).

To further understand the relationship between the structure of the Env-CT and its incorporation into the Gag lattice**,** we aimed to determine whether a monomeric transmembrane protein possessing a single Env-CT was sufficient for trapping and retention in virus assembly sites using a Gag MA-dependent mechanism. Previous work has demonstrated that a chimeric gp41 comprised of green fluorescent protein (GFP) fused to a truncated gp41 ectodomain, the gp41 TMD, and the Env-CT can be incorporated into virus-like particles (VLPs); however, no validation of the monomeric character of the probe was performed in this previous study (24). Furthermore, this GFP-gp41 chimera was never shown to be dependent upon the Gag MA domain. To determine whether only the Env-CT is sufficient for viral lattice trapping, we generated A2.01 T-cell and COS7 cell lines (both CD4-null) stably expressing host-cell derived ecto- and transmembrane domains of CD4 fused to the Env-CT. CD4 is biosynthesized and functions as a monomeric type-I transmembrane protein but has the potential to oligomerize on the plasma membrane (25, 26). To eliminate the oligomerization potential of the CD4-EnvCT chimeras, we introduced a previously validated mutation, 318KE, into the CD4 ectodomain to produce monomeric CD4 (mCD4) (26). We additionally introduced the 712YA mutation into the Env-CT, a motif known to abrogate endocytosis of Env (27–29) and increase surface expression on the plasma membrane (11). We have previously shown, using single-molecule imaging of Env-712YA, that this residue is not required for lattice trapping in the context of the native Env trimer (11); however, this does not discount the role of this residue for regulating vesicular trafficking of Env. An Env-CT null (mCD4-ΔCT) version of the chimera was generated to control for nonspecific interactions between the CD4 transmembrane domain and virus assembly sites (Fig. 1A).

To confirm that our mCD4-EnvCT chimeras are predominantly monomeric on the plasma membrane, we utilized orthogonal biochemical and biophysical approaches. We first chemically cross-linked stably expressed mCD4-EnvCT exposed on the plasma membrane using bis(sulfosuccinimidyl)suberate (BS^3^). No higher molecular weight species were observed in these cross-linking experiments for mCD4-EnvCT stable cell lines, while native CD4-expressing cells produced a higher molecular weight CD4 positive band as visualized by western blot (Fig. S1A). Next, we imaged single molecules of mCD4-EnvCT, mCD4-∆CT, and native CD4 using direct stochastic optical reconstruction microscopy and performed quantitative cluster analysis to estimate the extent of oligomerization of these proteins on the plasma membrane (30). Density-based spatial clustering of applications with noise (DBSCAN) (31) and Gaussian mixture model analysis of super-resolved chimeras and native CD4 revealed oligomeric-like clusters for CD4 (75% monomeric) and a less pronounced clustered species for mCD4-EnvCT chimeras (90% monomeric) (Fig. S1B-F). In all, these results suggest that nearly all chimeric mCD4-EnvCT resides on the plasma membrane in a predominantly monomeric state.

We also designed an orthogonal synthetic Env-CT chimera, consisting of an HA epitope-fused HaloTag ectodomain linked to the transmembrane domain of low-density lipoprotein receptor (LDL) (32, 33). The C-terminus of this synthetic type-I integral membrane receptor is fused to either the Env-CT (possessing the 712YA mutation) or an Env-CT truncation mutant, to create HA-Halo-gt-EnvCT and -∆CT, respectively (Fig. S2A). To validate the monomeric character of the Halo-gt-EnvCT construct, we synthesized a bifunctional halo-tag ligand (BiHTL) to site specifically cross-link molecules *in-cellulo* (Fig. S3). We performed crosslinking on COS7 cells stably expressing HA-Halo-gt-EnvCT at 4°C and 25°C to control for lateral diffusion of chimeras on the plasma membrane. The assay produced little appreciable crosslinking for EnvCT chimeras at 4°C compared to non-crosslinked controls (Fig. S2B-C), further supporting the monomeric character of these orthogonal chimeras.

### Gag-lattice trapping is EnvCT-dependent, leading to strong immobilization of monomeric chimeras within virions

To determine whether mCD4-EnvCT could be incorporated into VLPs in an Env-CT-dependent manner, we transduced A2.01 mCD4-EnvCT and -ΔCT stable cell lines with VSV-G pseudotyped HIV-1 NL4-3 *pol*(-), *env*(-) virus. VLPs produced by A2.01 cells stably expressing either mCD4-EnvCT showed ~16.7-fold elevated levels of incorporation of the Env-CT relative to the ΔCT chimera, indicating a strong incorporation preference for Env-CT-containing monomers at virus assembly sites (Fig. 1B). While these results demonstrate an Env-CT dependence for the mCD4-EnvCT chimera-expressing cell lines, it is possible that the chimera dwells in the lattice long enough to become passively incorporated through weak or non-specific interactions with the viral membrane (15, 34–38). To test this possibility, we performed BiHTL chemical crosslinking of HA-Halo-gt-EnvCT chimeras on purified immature virus particles, we reasoned that weak, non-specific interactions would allow for diffusion of EnvCT chimeras leading to HA-Halo-gt-EnvCT crosslinking (Fig. S4). Virus particles were treated with BiHTL and crosslinked chimeras were quantified by western blot densitometry. HA-Halo-gt-EnvCT showed no increase in cross-linking compared to non-cross-linked virus (Fig. S4). These results collectively suggest that Env-CT chimeras largely exist as monomers on the plasma membrane, accessing the Gag lattice independently, and oligomerization is not a prerequisite for virus incorporation. Furthermore, Env-CT monomers are either trapped at virus assembly sites by directly interacting with the Gag MA layer or through molecular crowding on the immature virus particle. To further evaluate these possibilities, we sought to measure the retention of monomeric Env-CT in the lattice by diffusion of single molecules and membrane-independent co-sedimentation.

### A monomeric Env-CT chimera is retained in the immature HIV-1 Gag lattice upon detergent stripping

**Fig 2:**
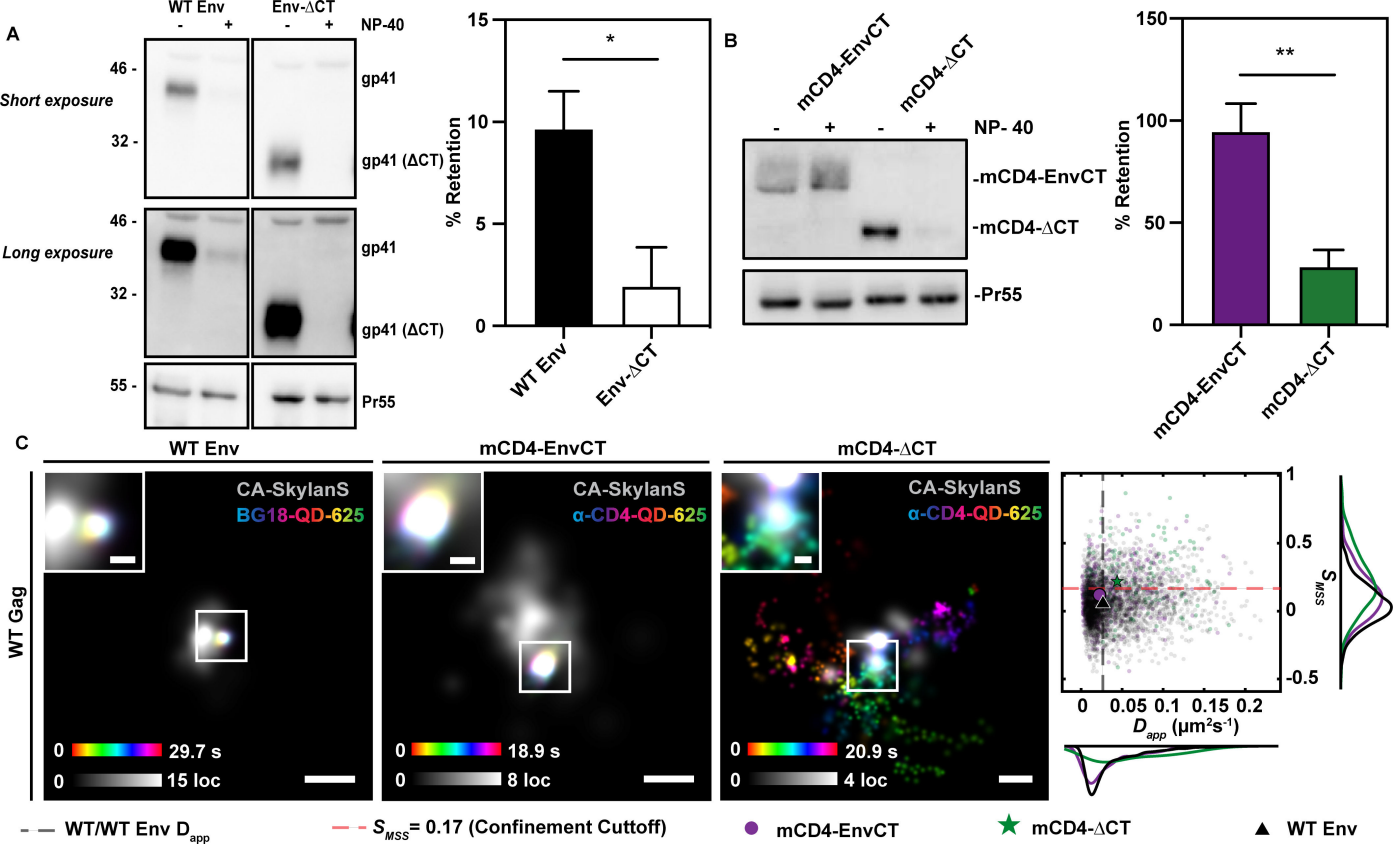
A monomeric Env-CT chimera is retained in the immature HIV-1 Gag lattice. (A, B) HEK293T cells were co-transfected with pNL4-3 Δ*pol*Δ*env* and mCD4-EnvCT or mCD4-ΔCT chimera plasmids. VLPs containing HIV-1 WT Env or EnvΔCT were produced using pNL4-3 Δ*pol* or pNL4-3 Δ*pol* CTdel144. After 48 hours, the virus was harvested, and membranes were stripped using NP-40 detergent. Samples were immunoblotted for Env (10E8v4/2F5), CD4 (ab133616), and Pr55 (HIV-Ig). Approximately 10% of WT Env is shown to co-sediment with Gag (Pr55) after detergent treatment, whereas Env-ΔCT trimers are stripped from immature VLPs (**A**) (*P* = 0.025, *n* = 4). Similarly, mCD4-EnvCT monomers co-sediment with Gag while mCD4-ΔCT is largely stripped from immature VLPs. (**B**) (*P* = 0.0066, *n* = 4). (**C)** WT COS7 cells (left) or COS7 cells stably expressing mCD4-EnvCT (middle)/mCD4-∆CT (right) were infected with NL4-3 virus (∆*pol*/∆*vif*/∆*vpr*/±*env/*∆nef::anti-CA-SkylanS). After 38–42 hours, cells were labeled with anti-Env-QD625 or anti-CD4-QD625 conjugates and imaged using TIRF microscopy at 37°C and 5% CO_2_. Gag was detected by coexpression and imaging of anti-CA nanobody fused to the photoswitchable SkylanS fluorescent protein. Time projections of individual single-particle tracks (color bar) proximal to superresolved HIV-1 assembly sites (gray). Individual tracks display confined or Brownian mobility dependent on the presence or absence of an Env-CT, respectively (scale bars = 200 nm, insets = 50 nm). Gray scale bars indicate Gag localization (loc) density over the time of acquisition, and color bars indicate particle position over the observation period. Confined tracks (mCD4-EnvCT, WT Env) appear white and centered in the insets. Scatter histograms of diffusion parameters: *D_app_* and *S_MSS_* for WT Env and chimera monomers with respect to WT Gag lattice assembly sites indicate a significant increase in mobility for mCD4-ΔCT as compared to mCD4-EnvCT and WT Env (mCD4-EnvCT: *D_app_* = 0.022 ± 5.7×10^−4^ µm^2^s^−1^, *S_MSS_* = 0.12 ± 0.18; mCD4-ΔCT: *D_app_* = 0.044 ± 1.6×10^−3^ µm^2^s^−1^, *S_MSS_* = 0.22 ± 0.22; WT Env: *D_app_* = 0.027 ± 1.9×10^−4^ µm^2^s^−1^, *S_MSS_* = 0.056 ± 0.16; mCD4-EnvCT, WT Env: *P_Dapp_* = 2.7 × 10^−4^, *P_SMSS_* = 1.2 × 10^−13^). The red dashed line, *S_MSS_* >0.17, indicates Brownian motion. The black dashed line represents the mean *D_app_* for WT Env. All errors represent SD.

Previous work has suggested that cellular host proteins and lipids may contribute to HIV-1 virion assembly; however, *in vitro* studies have provided evidence for a MA and Env-CT interaction in the absence of other protein and lipid influences (14, 15, 34–37, 39–41). Treating HIV-1 virions with detergent removes the viral membrane, yet, following NP-40 detergent treatment, HIV-1 Env co-sediments with the Gag lattice of immature VLPs in an EnvCT-dependent manner (42). To investigate whether our mCD4-EnvCT chimeras interact with the immature Gag lattice, we stripped viral membranes from immature VLPs, produced in HEK293T cells, containing either mCD4-EnvCT or mCD4-∆CT. As with native HIV-1 Env (Fig. 2A), we observed co-sedimentation of mCD4-EnvCT with Gag (Fig. 2A and B). Both HIV-1 Env-ΔCT and mCD4-ΔCT were significantly depleted from virus particles when treated with NP-40, indicating that mCD4-EnvCT retention in the immature Gag lattice is EnvCT-dependent. (Fig. 2A and B). These findings suggest that a monomeric Env-CT is sufficient to interact with the immature Gag lattice. We did observe the passive incorporation of mCD4-ΔCT into VLPs when transfected and overexpressed in HEK293T cells; however, mCD4-ΔCT is not incorporated into VLPs when stably expressed in A2.01 T-cells. This effect has been previously documented in cell lines deemed permissive, or EnvCT-independent, where high plasma membrane expression drives passive incorporation (43).

Next, we aimed to observe and compare the dynamics of single Env trimers and mCD4-EnvCT chimeras at virus assembly sites on live infected cells. We and others have previously demonstrated that Env is confined to sub-100 nm regions at virus assembly sites on the plasma membrane (8, 11–13, 35). The relegation of Env to sub-viral regions suggests the existence of a strong and localized retention mechanism between the MA lattice and the membrane-proximal Env-CT. To test whether a monomeric Env-CT is sufficient for sub-viral confinement, we performed single-molecule tracking of stably expressed mCD4-EnvCT in HIV-1-transduced (*pol*(-)/*env*(-)) COS7 cells and compared the diffusion behaviors to native Env trimers at virus assembly sites. We observed that mCD4-EnvCT and native Env trimers displayed similar confinement and retention at virus assembly sites (Fig. 2C). The apparent two-dimensional diffusion coefficient (*D_app_*) of mCD4-EnvCT trajectories (mean *D_app_* = 0.022 ± 5.7 × 10^−4^ µm^2^s^−1^) was slightly lower than that of WT Env (mean *D_app_* = 0.027 ± 1.9 × 10^−4^ µm^2^s^−1^) at assembly sites (*P_Dapp_* = 2.7 × 10^−4^). Furthermore, the slope of the moment scaling spectrum (*S_MSS_*), a measure of the frequency of sampling molecular positions within a given area, was higher for mCD4-EnvCT trajectories (*S_MSS_* = 0.12 ± 0.18) when compared to WT Env (*S_MSS_* = 0.056 ± 0.16), suggesting that, while highly confined to a sub-viral region, the monomer may have more freedom of movement within this region of the MA lattice compared to a trimeric Env-CT (*P_SMSS_* = 1.2 × 10^−13^). To then test whether the sub-viral confinement of mCD4-EnvCT molecules is dependent on the Env-CT, we performed single-molecule tracking of stably expressed mCD4-ΔCT on the surface of HIV-1-transduced (*pol*(-)/*env*(-)) COS7 cells. We observed mCD4-ΔCT trajectories with increased mean *D_app_* (0.044 ± 1.6 × 10^−3^ µm^2^s^−1^) and *S_MSS_* (0.22 ± 0.22) compared with mCD4-EnvCT, indicating that mCD4-ΔCT is unconfined and diffuses through Gag lattices (*P_Dapp_* = 4.8 × 10^−11^, *P_SMSS_* = 1.2 × 10^−7^). To quantify the degree of lattice confinement for mCD4-EnvCT versus WT Env using an orthogonal analysis method, we measured the full width at half maximum (FWHM) of all single-molecule trajectory positions residing within assembly sites. Both mCD4-EnvCT and WT Env displayed similar FWHM values (Fig. S5A and B). These results collectively suggest that, similar to native Env trimers, a monomeric Env-CT is sufficient to sense and become trapped by the underlying Gag lattice.

### A monomeric Env-CT is sensitive to perturbations of the Gag MA lattice

**Fig 3:**
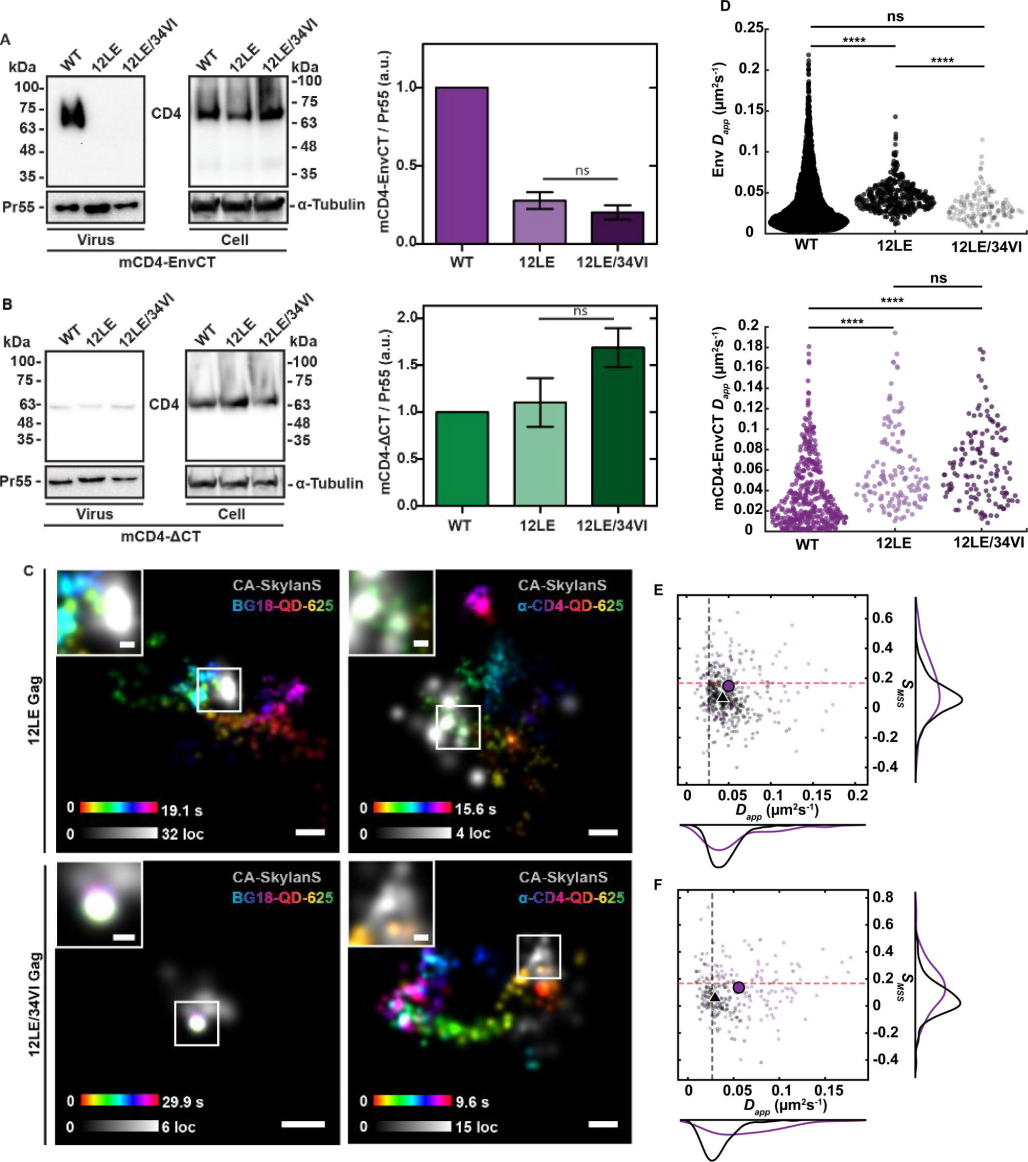
A monomeric Env-CT is sensitive to perturbation of the Gag MA domain. (A, B) A2.01 T-cells stably expressing mCD4 ± EnvCT were infected with a single-round infectious virus possessing the indicated Gag genotypes (NL4-3-∆*pol*/∆*vif*/∆*vpr*/∆*env*). After ~48 hours, virus supernatants and cells were harvested and subjected to Western blotting.** (A)** The 12LE and 12LE/34VI MA lattice are defective for mCD4-EnvCT incorporation. (**B)** mCD4-∆CT chimeras are insensitive to the 12LE and 12LE/34VI MA mutant lattice and passively incorporate regardless of the MA genotype.** (C)** COS7 cells stably expressing mCD4-EnvCT were infected with a single-round infectious virus possessing the indicated Gag genotypes (NL4-3-∆*pol*/∆*vif*/∆*vpr*/±*env/*∆nef::anti-CA-SkylanS). After 38–42 hours, native Env trimers were labeled with anti-Env (BG18)-QD625 (left panels) or mCD4-EnvCT molecules were labeled with anti-CD4-QD-625 conjugates (right panels) and imaged by TIRF microscopy at 37°C with 5% CO_2_. Reconstructions of Gag assembly sites were performed by single molecule sampling of expressed anti-CA nanobody fused to the photoswitchable SkylanS fluorescent protein. Representative time projections of single molecule trajectories for WT Env and mCD4-EnvCT diffusion in 12LE and 12LE/34VI MA lattices (mCD4-EnvCT: *D_app_* = 0.049 ± 1.3×10^−3^ µm^2^s^−1^, *S_MSS_* = 0.15 ± 0.19; WT Env: *D_app_* = 0.043 ± 3.9×10^−4^ µm^2^s^−1^, *S_MSS_* = 0.067 ± 0.12, *P_Dapp_* = 1.1 × 10^−3^, *P_SMSS_* = 3.4×10^−8^). Diffusion in 12LE/34VI lattices (mCD4-EnvCT: *D_app_* = 0.056 ± 1.4×10^−3^ µm^2^s^−1^, *S_MSS_* = 0.14 ± 0.17; WT Env: *D_app_* = 0.029 ± 6.2×10^−4^ µm^2^s^−1^, *S_MSS_* = 0.058 ± 0.14, *P_Dapp_* = 3.6×10^−17^, *P_SMSS_* = 7.5×10^−5^). Scale bars = 200 nm, inset = 50 nm. (**D)** Apparent diffusion coefficients for WT Env (top panel) and mCD4-EnvCT monomer (bottom panel) for all virus assembly sites measured. WT Env (BG18-QD-625) confinement is defective in the 12LE MA lattice; however, WT Env becomes diffusionally confined with the 12LE/34VI MA lattice rescue mutation (WT MA, 12LE: *P_Dapp_* = 2.9×10^−23^; 12LE, 12LE/34VI: *P_Dapp_* = 3.6×10^−17^; WT MA, 12LE/34VI: *P_Dapp_* = 0.16). Diffusional confinement is not rescued in the 12LE/34VI lattice for mCD4-EnvCT monomers (WT MA, 12LE: *P_Dapp_* = 3.7×10^−15^; 12LE, 12LE/34VI: *P_Dapp_* = 0.14; WT MA, 12LE/34VI: *P_Dapp_* = 8.5×10^−19^). (**E, F)** mCD4-EnvCT and WT Env have differing diffusion modalities in both the 12LE and 12LE/34VI mutant MA lattices, shown by *S_MSS_* and *D_app_* in scatter-histograms. The red dashed line indicates S_MSS_ >0.17, Brownian motion cutoff. The black dashed line indicates the mean *D_app_* for WT Env/MA diffusion. Black triangles are mean WT Env and magenta circles are mean mCD4-EnvCT values. All errors represent S.D.

Next, we aimed to determine whether the chimeric Env-CT monomer exhibits a dependence on MA, as has been shown for native Env trimers (11, 14, 15, 44, 45). To examine this, we introduced the MA-12LE mutation into our HIV-1 *pol*(-)/*env*(-) construct; this mutation abolishes Env incorporation and virus infectivity in the context of the fully infectious provirus but does not influence virus particle assembly (46). Incorporation of the mCD4-EnvCT chimera into the Gag lattice was markedly diminished by the MA-12LE mutation (Fig. 3A), whereas the low levels of mCD4-ΔCT passively incorporated into released particles were insensitive to the MA mutation (Fig. 3B). Next, we attempted to rescue incorporation of the mCD4-EnvCT chimera into MA-12LE particles by introducing the previously described 34VI mutation, known to rescue WT Env incorporation (45). Surprisingly, the incorporation of mCD4-EnvCT into virus particles containing the MA-12LE mutation could not be rescued by the introduction of the MA-34VI mutation (Fig. 3A). As expected, mCD4-ΔCT incorporation was unaffected by the MA mutations (Fig. 3B). Together, these findings suggest that a monomeric Env-CT is sufficient for virus particle incorporation in both a CT- and MA-dependent manner.

To better understand the mechanistic basis for MA dependence of the mCD4-EnvCT chimera, we examined its diffusion behavior when in proximity to mutant MA-12LE virus assembly sites. Consistent with our previous study (11), WT Env trimers can diffuse through virus assembly sites possessing Gag MA-12LE, avoiding the apparent confinement forces that lead to incorporation (mean *D_app_* = 0.043 ± 3.9×10^−4^ µm^2^s^−1^, *S_MSS_* = 0.067 ± 0.12) (Fig. 3C through E). We and others have suggested that this lack of confinement and incorporation in the MA-12LE lattice is mechanistically linked to a rearrangement of the MA lattice architecture (11, 14, 47). Notably, mCD4-EnvCT proximal to sites of MA-12LE assembly displayed an increase in mobility, similar to WT Env (mean *D_app_* = 0.049 ± 1.3×10^−3^ µm^2^s^−1^, *S_MSS_* = 0.15 ± 0.19) (Fig. 3C through E), indicating that the MA-12L residue contributes to confinement of monomeric Env-CTs (*P_Dapp_* = 1.1 × 10^−3^, *P_SMSS_* = 3.4 × 10^−8^).

Next, we sought to determine why the MA-34VI rescue mutation was unable to facilitate the incorporation of mCD4-EnvCT using single-molecule tracking at virus assembly sites. We observed that MA-34VI restores confinement for Env trimers at MA-12LE virus assembly sites (mean *D_app_* = 0.029 ± 6.2×10^−4^ µm^2^s^−1^, *S_MSS_* = 0.058 ± 0.14); however, mCD4-EnvCT showed elevated mobility and sampling of lattice positions in the presence of this rescue mutation (mean *D_app_* = 0.056 ± 1.4×10^−4^ µm^2^s^−1^, *S_MSS_* = 0.14 ± 0.17) (Fig. 3C, D and F). This result suggests that an Env-trimer-specific lattice retention mechanism was selected with the MA-34VI rescue mutation (45, 46). Given the recent structure of the ordered trimeric Env-TMD and -CT (23), our results suggest that the Env-CT baseplate is not important for Env retention in the WT MA lattice; however, compensatory mutations such as 34VI may organize the MA lattice to accommodate quaternary structural features in the baseplate.

### Monomeric Env-CT competes with native Env trimers for virion incorporation

**Fig 4:**
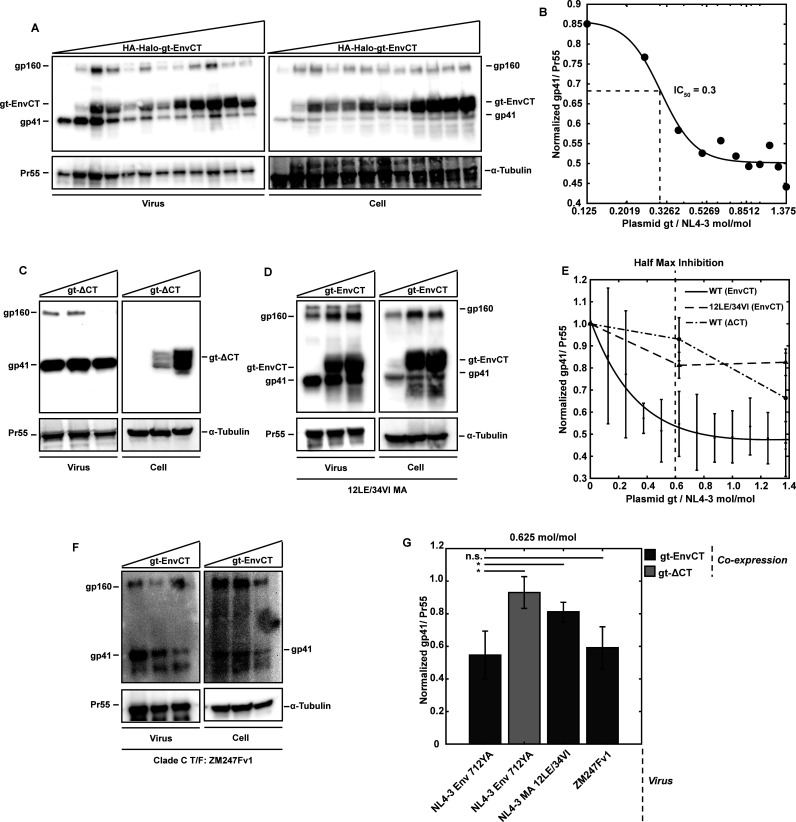
Monomeric Env-CT competes with native Env trimers for virion incorporation. Env incorporation competition experiments were performed by transfecting HEK293T cells with plasmids for single-round infectious HIV-1 (NL4-3- or ZM247Fv2-∆pol/∆vif/∆vpr) and HA-Halo-gt-EnvCT(712YA) chimera. (**A)** Representative western blots of VLPs and cell lysates for competitive inhibition experiments using Env 712YA with increasing plasmid concentrations of monomeric HA-Halo-gt-EnvCT(712YA). (**B)** A dose-response curve was estimated using a sigmoidal fit (IC_50_ = 0.3 mol_gt_/mol_NL4-3_; mean square error = 1.2 × 10^−3^). (**C)** Western blots demonstrate that HA-Halo-gt-∆CT does not compete with Env 712YA with increasing plasmid ratios (0, 0.625, and 1.375 mol_gt_/mol_NL4-3_). (**D)** Western blots showing monomeric HA-Halo-gt-EnvCT cannot inhibit WT Env incorporation in the 12LE/34VI mutant MA lattice. For all western blots, gp41 (Env 712YA) and HA-Halo-gt-EnvCT were detected with anti-EnvCT (Chessie8) mAb, Gag was detected with anti-p24 (183-H12-5C) mAb, and the HA epitope with anti-HA (**H17-L2**) mAb. (**E)** Double exponential loss curve of the competitive inhibition of 712YA Env with half-maximal inhibition occurring at 0.6 ± 0.1 gp41/Pr55 (vertical dashed line represents half-maximum of inhibition; root mean square error of fit = 0.04). (**F)** Clade C transmitted founder virus Env competition for virus assembly sites is observed with expression of HA-Halo-gt chimera possessing the Clade B (NL4-3) Env-CT. (**G)** Comparisons of the mean gp41/Pr55 ratio between all assays performed at 0.625 mol_gt_/mol_NL4-3_ plasmid ratios (MA-WT/Env-712A versus MA-WT/gt-∆CT: *P* = 0.02; MA-WT/Env-712YA versus MA-12LE-34VI/gt-EnvCT: *P* = 0.04; MA-WT/gt-∆CT versus MA-12LE-34VI/gt-EnvCT: *P* = 0.15, MA-WT/Env-712YA versus ZM247Fv1/gt-EnvCT: *P* = 0.69). Error bars represent SD.

Our results demonstrate that mCD4-EnvCT phenocopies the dependence on the MA-12L residue for trapping and retention when compared to native Env trimers. We next sought to determine whether the regions of the MA lattice that promote Env-CT retention are identical or overlapping between monomeric Env-CT and native Env trimers. We designed a competition assay to determine whether monomeric Env-CT can saturate the MA lattice-interacting regions and restrict native Env trimer incorporation. Co-expression of mCD4 chimeras and WT Env would likely give rise to binding and clustering during biosynthesis, as the CD4 ectodomain interacts with Env for mediating HIV-1 entry (48–50). To ameliorate this issue, we used the validated monomeric HA-Halo-gt-EnvCT chimera construct to produce VLPs. HaloTag chimeras were co-transfected with NL4-3 *pol*(-) Env-712YA in a range of molar ratios from 0 to 1.375 mol/mol, respectively. VLPs incorporated both WT Env and Env-CT chimeras. Importantly, increased expression of monomeric HA-Halo-gt-EnvCT restricted incorporation of Env-712YA trimers in a dose-dependent manner (IC_50_ = 0.3 mol_HA-Halo-gt-EnvCT_/mol_NL4-3,WT-Env_) (Fig. 4A B, E, and G), similar to previous measurements with another Env-CT chimera (24). We determined that this result was dependent upon the Env-CT as the tailless HA-Halo-gt-ΔCT failed to inhibit trimeric Env-712YA incorporation (Fig. 4C, E, and G). Consistent with the observation that the monomeric Env-CT is not rescued by MA-12LE/34VI particles, no restriction of native Env incorporation was observed in this mutant background (Fig. 4D, E, and G), suggesting that Env-CT overexpression does not perturb upstream Env biosynthesis and trafficking.

We next sought to determine whether the HA-Halo-gt-EnvCT chimera, derived from the Env-CT of an HIV-1 Clade B molecular clone (NL4-3), could restrict native Env incorporation into viruses from another HIV-1 clade. To test this, we challenged an HIV-1 Clade C transmitted/founder (T/F) clone, ZM247fv1 (*pol*(-), *vif*(-), *vpr*(-)) (41), by coexpression with HA-Halo-gt-EnvCT (Clade B; NL4-3). We observed that HA-Halo-gt-EnvCT restricts the incorporation of Clade C ZM247fv1 Env, similar to that of Clade B (NL4-3) Env restriction (Fig. 4F and G). We also observed that the HA-Halo-gt-EnvCT monomer was unable to restrict the incorporation of murine leukemia virus Env-YFP into the NL4-3 Gag lattice (Fig. S6), an evolutionarily distant viral glycoprotein able to pseudotype HIV-1 particles (51). Finally, we show that HA-Halo-gt-EnvCT can itself be saturated on released particles (Fig. S7), similar to previous observations with native HIV-1 Env overexpression (52). Together, these results support a model where monomeric Env-CT chimeras occupy the native incorporation sites of Env trimers within the MA lattice, suggesting that the nature of the interaction between Env-CT and the MA layer may be relegated to a limited number of secondary or tertiary contact points that are structurally conserved across clades of HIV-1 species.

## DISCUSSION

A current barrier to understanding how HIV-1 Env glycoproteins are incorporated and retained at virus assembly sites is the lack of high-resolution structural information for the native components at virus assembly sites and within particles. In the absence of this structural information, we sought to determine the oligomerization requirements for the HIV-1 Env-CT and the Gag-MA layer. Our study suggests that the fundamental structure mediating Env-CT – Gag-MA coalescence is less complex than a quaternary surface and likely comprises a core secondary or tertiary conformation of monomeric Env-CT. By producing a chimeric Env-CT monomer, we have demonstrated that monomers of Env-CT are sufficient for virus particle incorporation, suggesting that the putative trimer baseplate structure may be unnecessary for the assembly process, yet be required for additional steps such as fusion and entry regulation.

Previously, we demonstrated that measuring diffusional confinement of HIV-1 Env trimers at sites of Gag lattice assembly can discern passive incorporation (pseudotyping) from *bona-fide* Env-CT retention in the viral lattice (8, 11, 12). In this study, we speculated that it would be possible for a monomeric Env-CT to have some preference for assembly sites in the absence of a quaternary structure yet be able to diffuse through the lattice without strong multivalent retention. Using our single-particle tracking methodology, we demonstrate that a monomeric Env-CT is sufficient for strong diffusional confinement at HIV-1 assembly sites, resembling that of native Env trimers. These results support a model where secondary or tertiary structures, from a monomeric Env-CT tail, are utilized for glycoprotein retention in the viral lattice. We cannot rule out avidity as being a mechanistic factor in Env trimer entrapment within the MA lattice; however, we observed similar single-molecule diffusion of native Env and our EnvCT chimeras at virus assembly sites.

Our study shows that monomeric Env-CT incorporation is also dependent on the Gag-MA layer, as perturbations to MA abrogate native Env incorporation (11, 14, 15, 44, 45). We found that the monomer is readily incorporated into released particles in an MA-dependent manner and phenocopies the increased trimer diffusivity in the defective MA-12LE budding Gag lattice. These observations suggest that the MA-12LE mutation permeabilizes the MA layer or abolishes structural complementarity, allowing for a monomeric Env-CT to diffuse through the lattice without retention. Introduction of the 34VI mutation into the MA-12LE lattice rescues Env trimer incorporation, yet it fails to retain the monomer, suggesting that 34VI may stabilize the lattice only enough for bulkier Env trimers to become corralled or, conversely, this rescue mutation may create an orthogonal interface specific for trimer retention. A recent study from the Barklis group investigated the consequences of perturbations to the Env-CT baseplate structure and found that a series of mutations in the TMD-proximal core of the baseplate structure leads to a threefold reduction in Env incorporation, which is rescued by the same 34VI mutation (53). These results suggest that the 34VI rescue mutation is not specific to the folded baseplate, but may act by relieving a diffusion barrier for entry of bulkier unfolded Env trimers, as we have suggested for similar deletion mutants of the Env-CT (8).

We have additionally shown that a monomeric Env-CT is saturable for particle incorporation, and this monomer can dose dependently restrict native Env trimer incorporation. These results suggest that a monomeric Env tail can only occupy a limited number of regions within a Gag lattice, and these regions are the same for monomers and trimers. Consistent with this hypothesis, HIV-1 particles are decorated with only 7–19 Env trimers (54, 55), despite having roughly a thousand predicted Gag MA trimers in the immature lattice that could potentiate Env incorporation. Furthermore, single-molecule tracking of Env trimers suggests that encounters between freely diffusing Env and Gag lattices are relatively frequent over the course of virus assembly (11, 12, 56, 57). If every MA trimer were able to interact with an Env-CT, there should be 100-fold more Env trimers per particle. Collectively, this suggests that heterogeneous MA lattice regions exist that accommodate both native quaternary and linear Env-CT tail structures, potentially within defects that evolve in the MA lattice structure (6, 58). This hypothesis is supported by a recent cryo-EM structure demonstrating that the immature MA lattice of HIV-1 particles is somewhat heterogeneous across the particle surface (59). Conversely, the incorporation mechanism may rely heavily on building a strong diffusion barrier, inhibiting entry of Env into the MA lattice, resulting in few trimers per particle and helping HIV-1 to evade antibody neutralization. Consistent with this hypothesis, we have observed the clustering of native Env at the neck of budding viruses, suggesting that Env associates more frequently with the edges of the Gag lattice (8). We speculate that perturbations to the baseplate structure exaggerate this barrier effect by unfolding the three tails and acting sterically to inhibit entry at the edges of the lattice; however, a monomeric Env-CT can overcome this barrier due to overall lower mass and access lattice internal sites for incorporation. It is intriguing to speculate that native Env trimers can undergo conformational changes between a folded quaternary baseplate structure and a more linear structure, with these conformers mediating distinct lattice entry and trapping mechanisms, respectively.

Finally, this work demonstrates that a clade B monomeric Env-CT can restrict the incorporation of a clade C T/F virus Env. This suggests that the mechanism of Env incorporation is conserved among divergent HIV-1 clades. By contrast, we did not observe incorporation restriction of the distant retroviral envelope glycoprotein from murine leukemia virus, suggesting that a selective and species-specific mechanism is utilized by HIV-1 Env for particle incorporation. These observations are critically important to the development of future incorporation antagonists because disruption of this Env-CT and Gag-MA interface may prove to be pan-HIV neutralizing. Overall, this work suggests that saturating this interface with Env-CT mimetics can restrict native Env incorporation into HIV-1 virions.

## MATERIALS AND METHODS

### Plasmid production

The monomeric 318KE mutant human CD4 isoform 1 ecto- and the transmembrane domain were created as a synthetic human codon-optimized gBlock (IDT). The synthetic construct included the entire CD4 ecto- and transmembrane domain up to residue 420V and was assembled into the pLJM1 lentiviral transfer plasmid for eukaryotic expression using the AgeI and EcoRI restriction sites (Addgene, plasmid #19319) (60). The NL4-3 712YA mutant Env-CT, beginning at residue 707R after the transmembrane domain, was fused to the CD4 TMD at the C-terminus to make mCD4-EnvCT. The mCD4-ΔCT construct was designed with the short C-terminal tail—GSWPAS (61) (Fig. 1A). The HIV-1 pSV-NL4-3 plasmids were designed as previously described (12). Briefly, the NL4-3 reference genome was cloned into an SV40 ori-containing backbone (pN1 vector; Clontech/Takara Bio USA). For chimera incorporation, the NL4-3 plasmids were modified by removal of the BclI-NsiI and NsiI-BglII fragments to delete *pol* and *env*, respectively. For competition experiments with Env(+) VLPs, 712YA *env* was produced by site-directed mutagenesis in a shuttle plasmid and was ligated into the NL4-3 backbone using the EcoRI and XhoI sites (11). For all imaging experiments, the NL4-3 genome was modified to prevent virus particle release by mutation of *gag* at residues 455–458 PTAP to LIRL, and deleted for *vif*/*vpr* by removal of the AflII-AflII fragment. All imaging constructs were additionally modified to include the CA-binding nanobody probe, CANTD-SkylanS, in the *nef* splice site (8, 11, 12, 62). MA mutations were introduced by site-directed mutagenesis at residues 12L and 34V in a shuttle containing the AatII-SpeI fragment of *gag*. The fragment was then ligated into the expression plasmid at these sites.

The HIV-1 subtype C transmitted founder virus plasmid, pZM247Fv1, was obtained from the NIH HIV Reagent Program, Division of AIDS, NIH; ARP-11941, contributed by Dr. Beatrice Hahn. To create a replication-incompetent and single-round infectious virus with this clone, a gBlock (IDT Technologies) was synthesized encoding the region between the ZM247Fv1 SpeI site in *gag* and the AfeI site in *tat,* and the *pol, vif,* and *vpr* sequences were removed. The polypyrimidine tract (cPPT) and central terminal sequence (CTS) from ZM247Fv1 were added back in the gBlock in place of ∆*pol/*∆*vif/*∆*vpr* to maintain transduction efficiency. This gene fragment was then PCR amplified and subcloned into the parental pZM247Fv1 plasmid using SpeI and AfeI restriction sites, restoring *gag* and *tat* open reading frames. The resulting pZM247Fv1-∆*pol/*∆*vif/*∆*vpr*-cPPT-CTS plasmid was used for single-round infectious virus production.

The HA-Halo-gt constructs were designed by linking the signal peptide (SP) of CD4 to the N-terminus of HA, the C-terminus of SP-HA was fused to the N-terminus of Halo Tag, the C-terminus of Halo Tag to a linker and N-linked glycosylation site (gt, LNGSKLQRPHQALGDVAGRGNEKKPSSVR)*,* and then the N-terminus of the LDL receptor transmembrane domain: ALSIVLPIVLLVFLCLGVFLLW. The gene cassette was codon optimized for mammalian expression and synthesized as a gBlock (IDT). The cassette was then inserted into the pLJM1 lentiviral transfer plasmid at AgeI and EcoRI restriction sites. A doxycycline-inducible lentiviral plasmid containing both the HA-Halo-gt-EnvCT and Env-ΔCT cassettes was generated by subcloning into the pCW57.1 vector (Addgene, plasmid #41393) between the NheI and AgeI restriction sites. The 712YA Env-CT was fused to the LDL receptor TMD beginning with Env residue 707R to make a monomeric Env-CT. The truncated Env-CT (ΔCT) was created with only the short anti-EnvCT Chessie 8 epitope (63) (GSSPDRPEG) fused to the C-terminus of the transmembrane domain (64). The MLV-YFP plasmid was described in Gregory, D.A. Lyddon, T.D., and Johnson, M.C. (65).

### Cells and cell culture conditions

The A2.01 CD4(-) and A3.01 T-cell lines were acquired through the AIDS Reagent Program, Division of AIDS, NIAID, NIH and provided by Dr. Thomas Folks (ARP-2059 and ARP-166, respectively) (66, 67) and cultured in RPMI (#17-105-CV, Corning) medium supplemented with 2 mM L-glutamine (#25-005-CI, Corning), 1 × penicillin-streptomycin (#30-003-CI, Corning), 1 × hypoxanthine (#25-047-CI, Corning), and 10% fetal bovine serum (FBS, #35–011-CV, Corning). A2.01 cells were transduced with pLJM1-mCD4-EnvCT/-ΔCT-derived lentivirus (see Virus production section below) and selected with 1 µg × mL^−1^ puromycin 48 hours post-infection. After 1 week, single cells were isolated and expanded. COS7 (CRL-1651) and HEK293T (CRL-3216) cells were acquired through ATCC (Manassas, VA) and cultured at 37°C with 5% CO_2_ in complete DMEM supplemented with 2 mM L-glutamine (#25-005-CI, Corning), 1 × penicillin-streptomycin (#30-003-CI, Corning), and 10% FBS (#35-011-CV, Corning). COS7 cells were selected for transduction with 1 µg × mL^−1^ puromycin for 1 week and cultivated in bulk as all cells were confirmed to express and display mCD4 chimeras. For the production of the doxycycline-inducible HA-Halo-gt-EnvCT/-ΔCT cell lines, COS7 were transduced with pCW57.1-derived lentivirus and selected with 2 µg × mL^−1^ puromycin at 48 hours post-transduction. No cell toxicity was observed at upwards of 12 µg/mL doxycycline at 48 hours post-induction.

### Virus production

Replication-incompetent lentivirus for both modified NL4-3, ZM247Fv1, and mCD4 chimera-containing constructs were produced through co-transfection of HEK293T with psPAX2 (Addgene, plasmid #12260) and pVSV-G (a gift from Dr. Xuedong Liu, University of Colorado, Boulder) plasmids with polyethyleneimine as previously described (11). Lentivirus was harvested 48 hours post-transfection and 0.45 µm filtered. VLPs were purified through a 20% sucrose cushion (#S7903, Sigma) in PBS by ultracentrifugation at 100,000 × *g* for 1 hour. For competitive inhibition experiments, HEK293T cells were transfected with both NL4-3 Env 712YA and HA-Halo-gt-EnvCT chimera plasmids. Cell and supernatant fractions were harvested 48 hours post-transfection.

### Western blotting

Incorporation assays were performed by single-round infection of A2.01-mCD4 cells. Supernatants from single-round infected (NL4-3 ∆Pol/∆Env) A2.01 or transfected HEK293T were harvested 48 hours post-transduction and -transfection, respectively, and clarified by 15,000 × *g* centrifugation. VLP fractions were isolated from clarified supernatant by ultracentrifugation at >100,000 × *g* over a 20% sucrose cushion using a Beckman SW-41ti rotor. Virus particle pellets were resuspended in phosphate-buffered saline (PBS). Cells were lysed with RIPA buffer (50 mM Tris, pH 7.4, 150 mM NaCl, 1% Triton X-100, 1 mM EDTA; Sigma Aldrich) supplemented with protease inhibitor cocktail (#S8830, Sigma-Aldrich). Cell lysates were clarified by centrifugation at 15,000 × *g* for 5 minutes. All samples were loaded onto SDS-PAGE 4%–20% gradient gels (#4568096, Bio-Rad) and transferred to PVDF membrane (#83-646R, Genesee Scientific). In the case of BiHTL sample preparation, cell lysates were resuspended in RIPA buffer containing 2% Triton X-100 and Laemmli gel loading buffer and boiled at 95° C for 10 minutes, passed through a 24-gauge needle 10 times, and resolved on a 10% SDS-PAGE gel. Membranes were blocked in PBST-M (PBS pH 7.4, 0.1% Tween-20, 5% non-fat milk wt/vol) for 1 hour at room temperature before primary staining under the conditions described below. After probing, membranes were washed thrice with PBST. Membranes requiring secondary stain were incubated in 5% PBST-M with secondary for 1 hour at room temperature and washed thrice with PBST. Membranes labeled with secondary horse-radish peroxidase (HRP)-conjugated antibodies were incubated in Pico Plus ECL (#20-300B, Genesee Scientific). Both HRP and fluorescence were detected using an imager (ProteinSimple FluorChem E Imager). The following antibody conditions were used in 5% PBST-M: anti-CD4 (Abcam, ab133616)—1:5,000 overnight 4°C; anti-p24—1:1,000 room temperature 1 hour; anti-gp41—1:1,000 overnight 4°C; anti-gp41 (10e8v4)—1:1,000 overnight 4°C; anti-alpha-Tubulin (clone B-5-1-2, #T5168, Sigma-Aldrich)—1:5,000 1 hour at room temperature; anti-Mouse HRP (#31430, Thermo Fisher)—1:10,000 1 hour room temperature; anti-Rabbit HRP (#AB_2307391, Jackson Immuno Research)—1:5,000 1 hour room temperature; Goat anti-human HRP (#62-8420, Invitrogen)—1:5,000 1 hour room temperature; rat anti-HA (clone 3F10; Roche)—1:5,000 overnight 4°C or 1:1,000 1 hour room temperature and goat anti-rat HRP (#31470, Invitrogen)—1:5,000 1 hour room temperature for competition experiments and BiHTL experiments, respectively; anti-gp41 (10E8/2F5) and anti-HIV-Ig (#ARP-3957)—1:10,000 overnight 4°C for detergent stripping assays. The anti-HIV-1 p24 monoclonal IgG was obtained through the NIH AIDS Reagent Program, Division of AIDS, NIAID, NIH from Dr. Bruce Chesebro and Kathy Wehrly (#ARP-6457) (68, 69). The polyclonal Anti-Human Immunodeficiency Virus Immune Globulin was obtained through the NIH AIDS Reagent Program, Division of AIDS, NIAID, NIH from Dr. Luiz Barbosa. The anti-HIV-1 gp41 10E8v4 monoclonal IgG was obtained through the NIH AIDS Reagent Program, Division of AIDS, NIAID, NIH from Dr. Peter Kwong (#ARP-12865)(70). The anti-HIV-1 gp41 2F5 monoclonal IgG was obtained through the NIH AIDS Reagent Program, Division of AIDS, NIAID, NIH (#ARP-1475). Chessie 8 was obtained through the NIH HIV Reagent Program, Division of AIDS, NIAID, NIH from Dr. George Lewis (#ARP-13049) (63). Densitometry was performed using ImageJ (FIJI) and analyzed in Matlab (Mathworks). The dose-response curve for the competitive inhibition assay (Fig. 4E) was generated using a sigmoidal fit of the mean normalized gp41/Pr55 densities with respect to the log_10_-transformed molar ratios of HA-Halo-gt-EnvCT to Env 712YA (WT). IC_50_ was determined to be the molar ratio at which normalized gp41/Pr55 decreases by one half.

### Membrane stripping

Membrane stripping assays were performed as previously described with some modifications (71). HEK293T cells were co-transfected with pNL4-3 Δ*pol*Δ*env* (72) and pLJM1-mCD4-EnvCT or pLJM1-mCD4-ΔCT using Lipofectamine 2000 (Invitrogen). VLPs containing HIV-1 WT Env or EnvΔCT were produced using pNL4-3 Δ*pol* or pNL4-3 Δ*pol* CTdel144 (45). VLPs were harvested 48 hours post-transfection and filtered through a 0.45 µm membrane. VLP preps were incubated with either 0.05% NP-40 for HIV-1 Env trimers and 0.075% NP-40 for mCD4 chimeras or PBS for 15 minutes at 25°C and then sedimented in a 20% sucrose cushion. VLPs were harvested at 60,000 × *g* at 4°C for 1 hour. The level of virion-associated gp41, mCD4-EnvCT, and Pr55 Gag in the pellets was determined by western blotting using anti-Env (10E8v4/2F5), anti-CD4 Ab (ab133616, Abcam), and HIV-Ig (Gag detection; ARP), respectively.

### Imaging probes

Mouse IgG1 anti-human CD4 clone SFCI12T3D11 (Beckman) was digested into Fab fragments using the papain proteolysis kit (#44985, Thermo Scientific). The purified Fabs were labeled with NHS-PEG4-Azide (#26130, Thermo Scientific) using a 1:1 molar ratio, free NHS-PEG4-Azide was removed using a 7K MWCO Zeba filter (#89883, Thermo Scientific), and Fab-PEG4-Azide was subsequently conjugated to the DIBO-QD625 quantum dot probe using copper-free click chemistry (Site-Click QD625 kit #S10452, Thermo Scientific). The resulting anti-CD4-QD625 fab conjugates were then purified from QD625 using CaptureSelect LC-kappa (murine) Affinity Matrix (#191315005, Thermo Scientific). Unconjugated anti-CD4 Fabs were removed using a 100 KDa MWCO protein concentrator (Site-Click kit), and anti-CD4-QD625 conjugates were concentrated to ~10 µM. The anti-gp120 HIV-1 Env BG18-QD625 was produced as previously described (12). Briefly, *p-azido-L-phenylalanine* modified BG18 Fab was recombinantly produced in *E. coli* by co-expression with an unnatural amino acid incorporation system (73). The fab was purified by multiple affinity resin matrices and then conjugated to DIBO-modified quantum dots using a site-specific copper-free click reaction (Site-Click QD625 kit). The conjugated fabs were further purified using a final CaptureSelect CH1-XL affinity matrix (#19434620, Thermo Scientific). The genetically encoded CA-SkylanS probe, an anti-CA camelid nanobody fragment fused to the reversibly switchable green fluorescent protein Skylan-S (74), was designed and expressed from the NL4-3 proviral genome as previously described (12).

### Fluorescence microscopy imaging conditions

COS7 cells were grown on fibronectin (FN)-coated coverslips and blocked in complete media supplemented with 10% BSA for 30 min at 37°C and 5% CO_2_. Cells were stained at room temperature with anti-CD4-QD625 or BG18-QD625 at ~10 nM final concentrations, respectively. Cells were washed with complete media thrice in intervals of 5 minutes. Cells were equilibrated at 37°C and 5% CO_2_ during image acquisition. High-speed acquisitions were streamed on a custom-built TIRF-M with isotropic field illumination as previously described (12). Briefly, images were acquired at 100 Hz using 50 mW of 473 nm laser power (measured at the rear aperture) for fluorescence excitation and images were collected using a 1.49 NA Nikon TIRF objective and Orca-Fusion Scientific-CMOS using standard scan mode (C14440-20UP Hamamatsu; Hamamatsu, Japan). The CA-SkylanS and QD625 probes were detected simultaneously by splitting the component channels into two halves of the camera using a W-View Gemini image-splitting mount (A12801-01 Hamamatsu). Image streams of a single cell typically consist of 1,000–3,000 images.

### Single-molecule tracking analysis

Analysis of tracking data was performed using custom Matlab (Mathworks) scripts as previously described (12). Briefly, single-molecule localizations and their precisions were determined using code modified from PeakSelector (https://github.com/gleb-shtengel/PeakSelector). Localizations from the respective CA-SkylanS and QD625 fluorescent channels were corrected for sub-pixel chromatic aberration using 100 nm fluorescent fiducials (TetraSpeck, T7279, Thermo Fisher Scientific) to register the channels for distance measurements between Gag assembly sites and single-molecule tracks. To determine the centroid of a single virus assembly site, CA-SkylanS localizations with positional uncertainty below 40 nm were binned into 50 nm pixels to generate a low-resolution probability density map (mean localization precision for CA-SkylanS σ = 30 ± 7 nm) (Fig. S5C). From this map, centroids were extracted based on maximum local intensity. Bona-fide Gag buds were determined to have a minimum of 10 localizations and <200 nm full-width at half maximum of binned localization positions. Centroids without these characteristics were excluded from the analysis. Localizations of quantum dots (average localization precision of σ = 11 ± 4 nm) (Fig. S5C) were passed to a custom software package written in Matlab for track linking (75) and tracks were sorted by their proximity to Gag lattice centroids (radius of influence = 125 nm, 10% of track localizations allowance outside the radius). Tracks determined to be proximal to virus assembly sites were then analyzed to compute mean squared displacement and the moment scaling spectrum previously described (11, 12, 76). Track diffusion coefficients and *S_MSS_* were pooled for statistical analysis using the inbuilt *ttest2* function in Matlab for log_10_(*D_app_*) and *S_MSS_* individually. The mean and associated error were extracted for log_10_(*D_app_*) and *S_MSS_* diffusion parameters by fitting them to normal distributions. The approximate full-width at half maximum (FWHM) estimates of probe displacements in the X and Y dimensions were extracted by first fitting individual tracks to normal distributions and subsequently calculating FWHM = 2.355 × σ, where σ is the standard deviation parameter extracted from the normal distribution fit. The X and Y FWHM estimates were averaged to generate one-dimensional histograms that were fit to lognormal distributions to extract mean and standard deviation.

## SUPPLEMENTAL MATERIAL

Supplemental material jvi.02105-24-s0001.pdf Supplemental methods; Figures S1 to S7.

ASM does not own the copyrights to Supplemental Material that may be linked to, or accessed through, an article. The authors have granted ASM a non-exclusive, world-wide license to publish the Supplemental Material files. Please contact the corresponding author directly for reuse.
