# Supplementary material for: A monomeric envelope glycoprotein cytoplasmic tail is sufficient for HIV-1 Gag lattice trapping and incorporation
Source: J Virol. 2025 Apr 15;99(5):e02105-24. doi: 10.1128/jvi.02105-24 (PMC12090761; doi:10.1128/jvi.02105-24)
Supplement: Supplemental material — Supplemental methods; Figures S1 to S7. [file jvi.02105-24-s0001.pdf]

Supplemental Material

A monomeric envelope glycoprotein cytoplasmic tail is sufficient for HIV-1 Gag lattice trapping and incorporation

Nicholas S. Groves[1], Austin R. Clark[1¶], Rebekah S. Aguilar[1¶], Yuta Hikichi[2], Anastasiia Kostenko[3], Merissa M. Bruns[1], Alegra T. Aron[3], Eric O. Freed[2], Schuyler B. van Engelenburg[1*]

[1] Molecular and Cellular Biophysics Program, Department of Biological Sciences, University of Denver, Denver, CO, 80210, USA
[2]Virus-Cell Interaction Section, HIV Dynamics and Replication Program, Center for Cancer Research, National Cancer Institute, Frederick, MD 21702-1201, USA.
[3] Department of Chemistry and Biochemistry, University of Denver, Denver, CO, 80210, USA
¶ These authors contributed equally to this study
* Correspondence to: schuyler.vanengelenburg@du.edu

Supplementary Methods.

Single-molecule localization microscopy sample preparation. Single molecule localization of endogenous CD4 was performed using #1.5 coverslips, containing gold nanorods as fiduciaries (#600-30AuF; Hestzig), coated with ICAM-1 (#552906, Biolegend) at a concentration of 2 ng×uL^{-1} for 30 min at 37°C. A3.01 (CD4 WT) were pelleted and resuspended in 2% methylcellulose (#M7027, Sigma-Aldrich) in complete RPMI media then added to coverslips and incubated at 37°C with 5% CO_2 for 45 minutes. For the chimeric COS7 stable lines, cells were grown overnight on #1.5 nanorod embedded coverslips that were coated with 20 µg×mL^{-1} human fibronectin (#FC010, Millipore). In all cases, the cells were fixed with 4% paraformaldehyde (PFA; #15700, Electron Microscopy Sciences) before blocking with 10% BSA (#A3983, Sigma-Aldrich) in DPBS (#14200-075, Thermo Fisher). Briefly, the anti-CD4 monoclonal antibody Ibalizumab (Trogarzo; Thera technologies) was directly conjugated to the AlexaFluor 647 dye using NHS chemistry, termed IBZ-AF647 (#A20006, Thermo Fisher). Antibodies have a degree of labeling of approximately 2. Staining of blocked cellular specimens was performed with 6% BSA in DPBS with IBZ-AF647 at a final concentration of ~12 nM. Samples were post-fixed with 4% PFA and 0.2% glutaraldehyde (#16020, Electron Microscopy Sciences) in DPBS for 1-2 min and quenched with 30 mM glycine (#G8898, Sigma-Aldrich) thrice for 5 minutes. Samples were then washed with a previously described STORM buffer and sealed in under an 18 mm coverslip with epoxy (8).

Single molecule localization microscopy and cluster analysis of mCD4-EnvCT chimeras. Single molecule localization microscopy was performed using the dSTORM method (30). Imaging was performed on a custom-built 4π TIRF microscope using only the bottom objective (1.49 NA Nikon apo TIRF) and a 640 nm laser (MRL-FN-640, OptoEngine) with 80 mW of excitation power projected onto the rear pupil of the objective (8). Fluorescence was filtered using 679/41 nm bandpass filter (FF01-679/41-25, Semrock). Images were acquired with an EMCCD camera (Andor DU-897; Oxford Instruments) using a 25 ms exposure, 5 MHz readout speed, and 200 electron multiplication gain. Localization of single molecule data was performed as described in the main methods except for a drift correction procedure using Au nanorod fiducials (#A12-25-600-CTAB-DIH-1-1, Nanopartz). Clusters analysis was performed with the inbuilt MATLAB function DBSCAN (31). The search radius for DBSCAN was set to 26.7 nm, equivalent to 2σ of our final localization precision. The minimum number of localizations in a cluster, set to 10 localizations, was determined empirically by assessing cluster assignment of spurious background outside of the cell perimeters. Each assigned cluster was fit to a 2D Gaussian distribution and the physical size of the cluster was estimated from the average of sigma x and sigma y parameters (assuming radial symmetry), The average sigma x/y was then

converted into a full width at half-maximum (FWHM) value. The distributions of cluster FWHM estimates were normalized and fit to both single and two term Gaussian functions using MATLAB's *fminsearch* multiparameter nonlinear local minimum solver (Figure S1B,C,D). Cumulative residual error for each fit was used as a metric for the presence of a second dimeric population for each genotype or chimera.

BS^3 Crosslinking.

Bissulfosuccinimidyl suberate (BS^3) (#A39266, Thermo Scientific) crosslinking was performed by resuspending cells (A2.01 mCD4-(EnvCT/ΔCT) or A3.01) to 25 million cells per mL in pH 8 DPBS and washed thrice. BS^3 was added to a final concentration of 3 mM and kept on ice for 30 minutes after which it was quenched with 1M Tris pH 7.4 to 20 mM and incubated for 15 minutes at room temperature prior to lysing in RIPA buffer containing 1% TX-100.

Production of Bivalent HaloTag Ligand (BiHTL).

One milligram of the bivalent HaloTag ligand (BiHTL) was produced by reacting one molar equivalent of HaloTag succinimidyl ester (O2) ligand (#P1691, Promega) with 1.5 molar equivalence of HaloTag amine (O4) ligand (#P6741, Promega) in dimethylformamide. The reaction was catalyzed using one drop of diisopropylethylamine (#D125806, Sigma) and allowed to proceed for 16 hours at room temperature with constant stirring. Both the HaloTag succinimidyl ester ligand and amine ligand were reacted at a 5 mg×mL^{-1} concentration.

HPLC Purification and UHPLC-MS Validation of Bivalent HaloTag Ligand (BiHTL).

The BiHTL reaction was diluted 10-fold in water. 100µL of diluted reaction mixture was loaded onto a Shimadzu Prominence-i LC-2039C 3D with Jupiter 5u C_{18} 300 Å 250×4.60 mm 5 µm column (Phenomenex). The mobile phase consisted of solvent A H_2O + 0.1% formic acid (FA) and solvent B acetonitrile (ACN). A linear gradient of solvent B from 0% to 50% was applied from 0 to 7 min, which was followed by another linear gradient to 99% solvent B from 7 to 20 min. Solvent A was held constant before subsequent sample injections to re-equilibrate the column. Separations were performed at room temperature and the product was detected at 200 nm. Peaks containing BiHTL were eluted at 79% ACN, pooled and analyzed by mass spectrometry.

For LC-MS/MS analysis, 2 µL of purified BiHTL was injected into a Vanquish UHPLC system coupled to a Q-Exactive orbitrap mass spectrometer (Thermo Fisher Scientific, Bremen, Germany). Data dependent acquisition (DDA) of MS/MS spectra was performed in positive mode. The MS scan range was set to 150–1500 m/z with a resolution of 30,000 at m/z 200 using a single micro-scan. The maximum ion injection time was set to 100 ms with an automated gain control (AGC) target of $1.0×10^6$. Up to five MS/MS spectra per MS1 survey scans were recorded in DDA mode with resolution $R_{m/z\ 200}$ 200 = 15,500 using a single micro-scan. The maximum ion injection time for MS/MS scans was set to 50 ms with an AGC target of $5×10^5$ ions. The MS/MS precursor isolation window was set to 1 m/z. Normalized collision energy was set to a stepwise increase from 25 to 35 to 45% with $z = 1$ as the default charge state. MS/MS scans were triggered at the apex of chromatographic peaks within 2–8 s from their first occurrence. Dynamic precursor exclusion was set to 5 s. Ions with unassigned charge states were excluded from MS/MS acquisition as well as isotope peaks. The data was analyzed in Thermo Xcalibur Qual Browser program. The m/z observed = 617.3326 with an m/z expected = 617.3335.

Temperature Dependence of BiHTL Crosslinking.

CW57.1-HA-Halo-gt-EnvCT/ΔCT COS7 cells were induced with 2 µg×mL^{-1} doxycycline (#D5207, Sigma-Aldrich) and assayed at 24 hours post-induction. Cells were removed from the dish with CellStripper (#25-056-CI, Corning) and resuspended in complete media. Upon resuspension, cells were treated with 5 µM BiHTL and reacted at 4° C or 25° C for 1 hour. At the one-hour timepoint, cells were immediately centrifuged down and resuspended in RIPA buffer containing 2% TX-100 then boiled in Laemmli gel-loading buffer.

BiHTL Crosslinking Reaction Conditions.

For virion BiHTL reactions, approximately 36 mL of ∆pol, ∆vif/vpr, ∆Env NL4-3 virus was produced in HEK293T and harvested at 48 h post transfection. COS7 cells were induced for HA-Halo-gt-EnvCT or -∆CT with 12 μg×mL^{-1} doxycycline and immediately transduced with virus. Fresh media and doxycycline were added to the COS7 cells 24 hours post-transduction. Virus from COS7 cells were harvested at 48-52 hours post-transduction, 0.45 μm filtered, and purified by ultracentrifugation (as described in main methods). Virus was resuspended in DPBS and reacted for 1 hour at 37° C with 500 nM BiHTL or DMSO carrier (#276855, Sigma-Aldrich) as control. The reaction was subsequently quenched by boiling in Laemmli buffer.

For cellular crosslinking BiHTL reactions, HA-Halo-gt-EnvCT or -ΔCT producing cells were infected with the aforementioned virus for 48 hours post-transduction. Cells were dissociated from the dish using CellStripper, washed in complete DMEM, resuspended in complete DMEM, and split for each reaction condition and controls. Cells were treated with or without 5 ng×uL^{-1} anti-HA IgG for 1 hour prior to crosslinking with 5 μM BiHTL for 3 hours at 4°C. Proteins were then extracted by lysing cells in cold RIPA buffer.

Supplementary Figures.

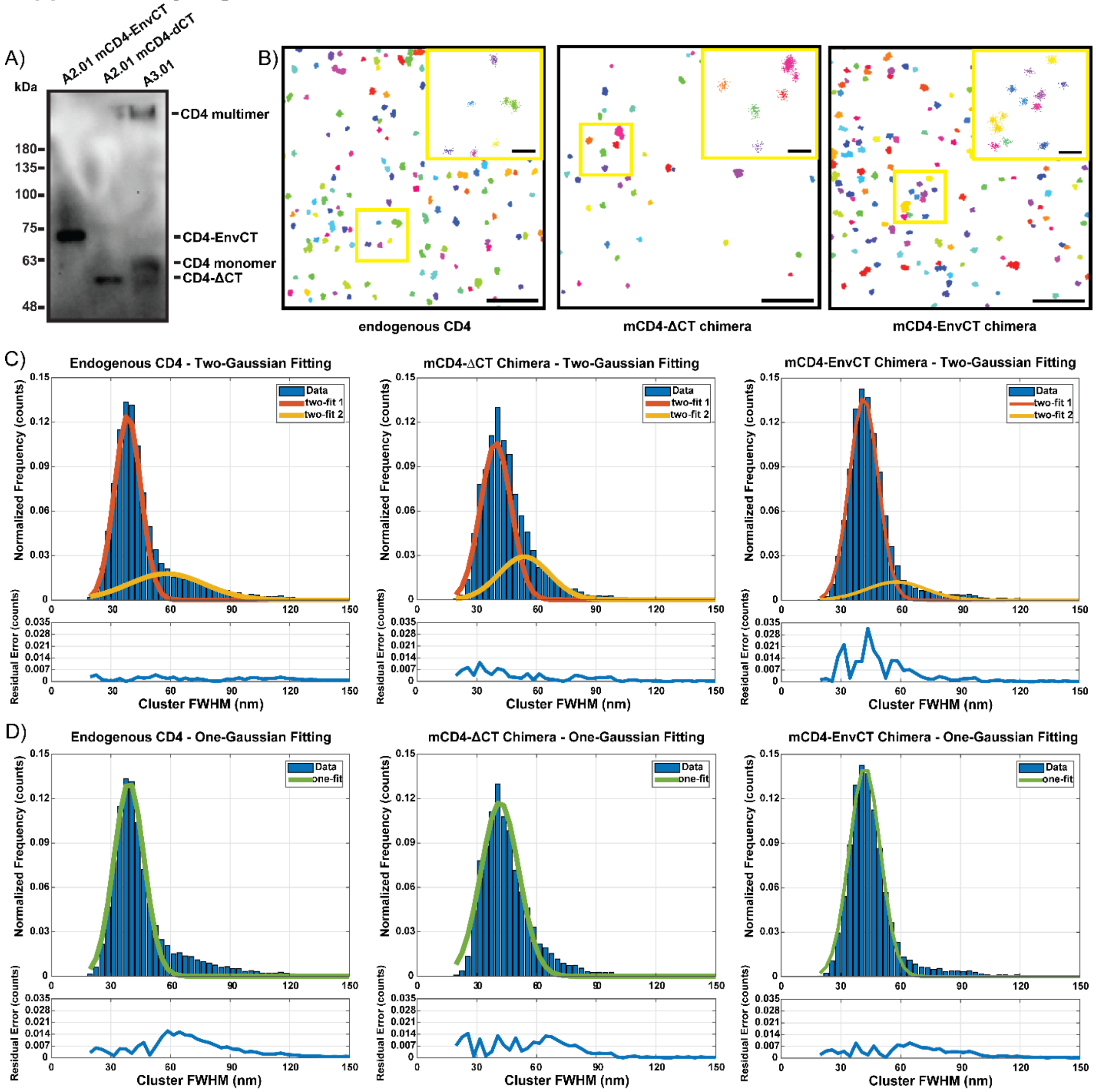

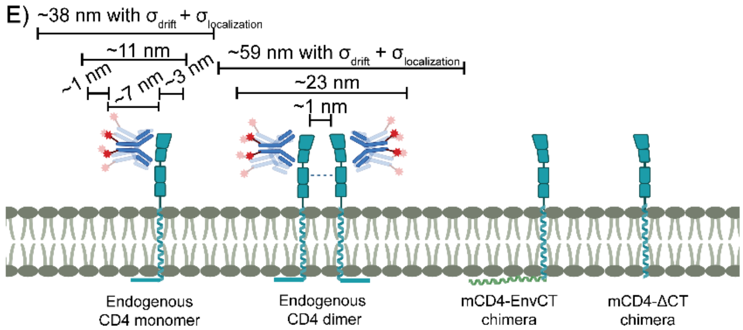

F)

Gaussian Fit:	One-fit (f_1)	Two-fit (f_2)	Difference (f_1 - f_2)
CD4	0.39	0.092	0.30
mCD4-dCT	0.38	0.21	0.17
mCD4-EnvCT	0.23	0.52	-0.29

Fig. S1. CD4(318KE)-EnvCT chimeras form stable monomers on expressing cells. A. Bissulfosuccinimidyl suberate (BS^3) crosslinking of A2.01 (CD4-null) cells expressing mCD4-EnvCT (left lane) or mCD4-EnvΔCT (middle lane) does not result in the accumulation of higher-

order oligomers, suggesting that both CD4-318KE and the Env-CT are incapable of driving multimerization of the chimeric constructs. The parental A3.01 cell line endogenously expresses CD4. BS^3 crosslinking of endogenous CD4 demonstrates that a population of CD4 exists in oligomeric forms (right lane; CD4 multimer), demonstrating that introduction of the 318KE mutation and removal of the CD4-CT relegates Env-CT chimeras to a monomeric form. Native CD4 and mCD4 chimeras were detected by western blotting with anti-CD4 antibody (ab133616, Abcam). **B.** Representative DBSCAN cluster analysis results from dSTORM imaging of endogenous CD4 (left) and COS7 cells stably expressing mCD4-ΔCT (middle) or mCD4-EnvCT (right). Scale bars are 1 μm and inset scale bars are 200 nm. **C.** Gaussian mixture model fitting of normalized cluster size (FWHM) distributions. Cluster distributions were analyzed from 9, 8, and 6 individual cells expressing endogenous CD4, mCD4-ΔCT, and mCD4-EnvCT, respectively. **D.** Single fit Gaussian model of normalized cluster size (FWHM) distributions. **E.** Model for expected physical dimensions of CD4 ectodomain and associated antibody probe. Residual error in drift correction (σ_{drift}) and our overall localization error ($\sigma_{localization}$) convolved with the size estimates of CD4 monomer and dimer populations. **F.** Cumulative residual error for single and two-Gaussian fits to each cluster size distribution. Similar residual error was observed for the two and single Gaussian fit to mCD4-ΔCT suggesting a small fraction of this probe may transiently exist as a dimer on the plasma membrane. The population of dimeric native CD4 was detected because the cumulative residual error was the lowest for a two Gaussian model fit compared to a single Gaussian model fit (0.092 versus 0.39). A higher overall residual error is observed for the two versus single Gaussian fit to mCD4-EnvCT size distributions, suggesting that the majority of EnvCT is monomeric on the plasma membrane.

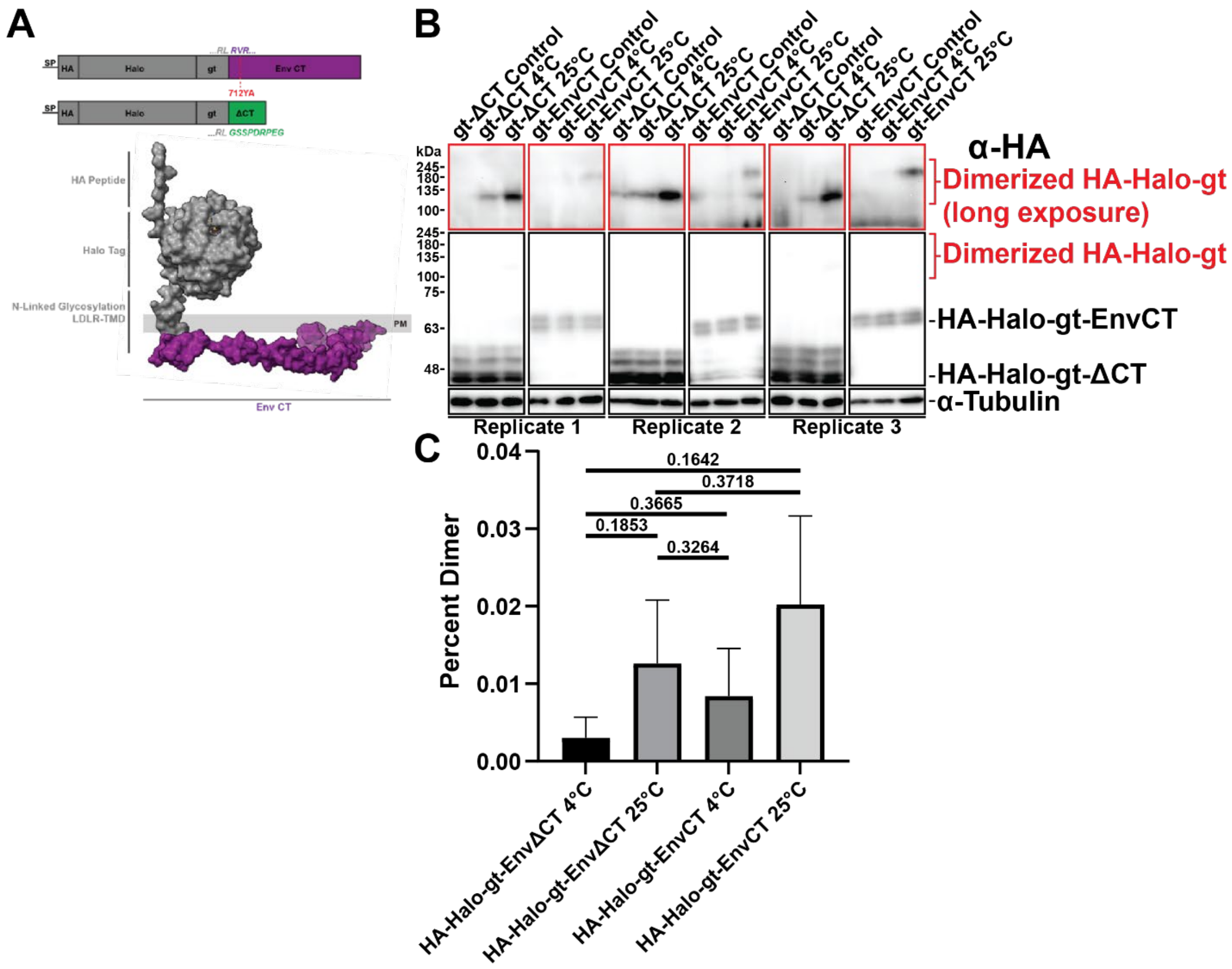

Fig. S2. HA-Halo-gt-EnvCT chimera design and temperature dependence of site-specific chemical crosslinking to assess dimerization potential. A. To remove the influence between the interaction with native Env trimers and the ectodomain of CD4, we designed an orthogonal monomeric Env-CT chimera. This alternative chimera utilizes a soluble HA-Halo tag ectodomain (gray, PDBID: 5Y2Y) with an N-terminal fusion to the CD4 secretory signal peptide (not depicted). This ectodomain was then fused to an N-linked glycosylation signal sequence and the transmembrane domain of the low-density lipoprotein receptor (LDLR-TMD). This was finally fused to either the full-length HIV-1 (NL4-3) Env-CT (magenta; PDBID: 5VWL) or to the short Chessie 8 peptide recognition region of Env-CT (green; ΔCT). **B.** COS7 cells stably containing tetracycline inducible chimeras were induced for 24 hours and subject to different temperatures and with or without the Bivalent Halo Tag Ligand (BiHTL) to chemically crosslink pre-existing chimera dimers. Replicate western blots demonstrating that the Bivalent Halo Tag Ligand (BiHTL) chemical crosslinking of the monomeric HA-Halo-gt-EnvCT is comparable to that of HA-Halo-gt-EnvΔCT. Crosslinking experiments were performed at two temperatures to control for diffusion and collisions between Halo constructs (reaction performed for 1 hour). This result suggests that crosslinking of these probes only occurs at 25°C due to surface diffusion and collisions, but not when molecular motion is restricted at 4°C. HA-Halo-gt-EnvCT/ΔCT were detected with anti-HA antibody. **C.** Densitometric analysis of the ratio of dimeric species relative to the total population. At 4°C, dimerization is observed at 0.84% for Env-CT and 0.30% for

ΔCT. At 25°C, the percentage dimerized increases to 2.02% for Env-CT and 1.26% for ΔCT. These results indicate that the Env-CT domain does not statistically enhance dimerization propensity (all P values > 0.05; two-tailed paired t-test). Error bars represent SD (n = 3).

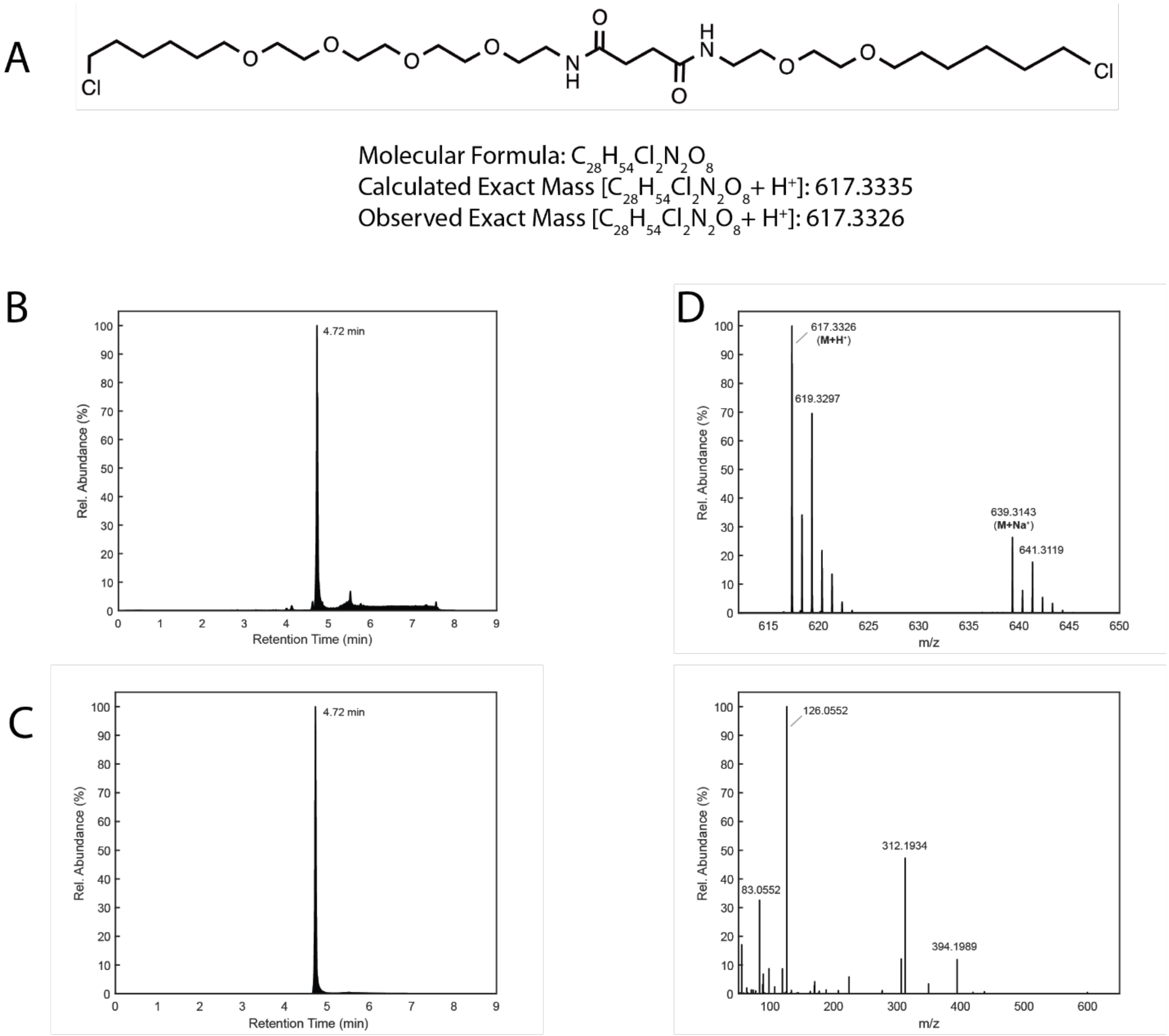

Fig. S3. Chromatographic profiles & mass spectra of Bivalent HaloTag Ligand (BiHTL). A. Chemical structure of BiHTL (N1-(18-chloro-3,6,9,12-tetraoxaoctadecyl)-N^4-(2-(2-((6-chlorohexyl)oxy)ethoxy)ethyl)succinamide). **B.** Total ion chromatogram (TIC) of HPLC fraction containing BiHTL. **C.** Extracted ion chromatogram (XIC) of mass 617.3326. D. LC-MS/MS spectra of BiHTL with MS1 spectrum (top) and MS2 spectrum (bottom).

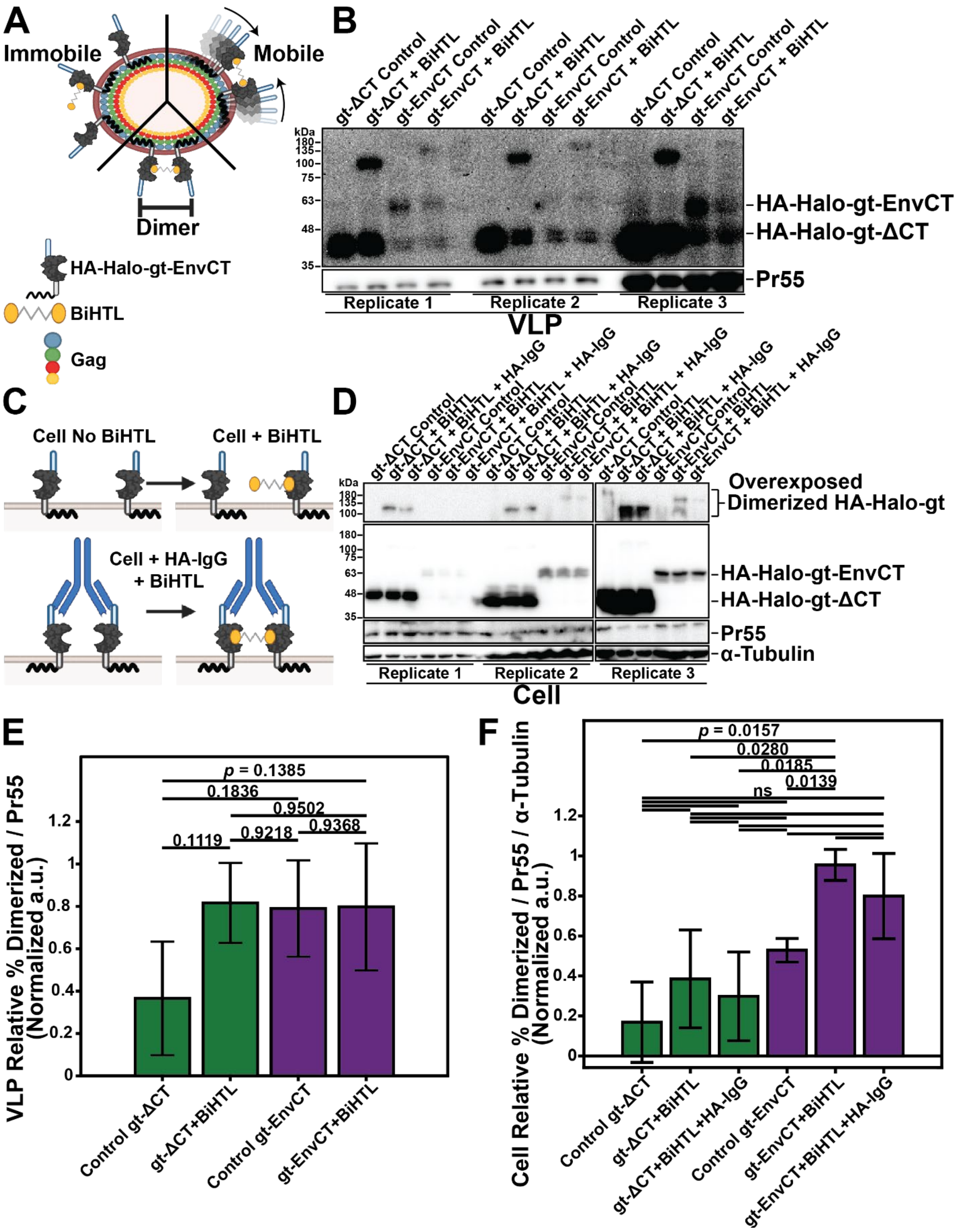

Fig S4. Site-specific chemical crosslinking of Halo Env-CT chimeras on VLPs demonstrates that monomers are predominantly incorporated and remain monomeric in released particles. COS7 cells, stably harboring an inducible HA-Halo-gt-Env±CT expression

cassette, were induced with 12 μg×mL^{-1} and infected with single-round infectious virus (NL4-3 Δ*pol*/Δ*env*). We note that the high expression driven by this artificial expression system leads to passive incorporation of ΔCT chimeras into VLPs. **A.** Model for BiHTL treatment of VLPs incorporating Halo-EnvCT chimeras: (i) immobile within the immature lattice, (ii) diffusion within the lattice leading to BiHTL crosslinking, and (iii) pre-existing oligomers of EnvCT chimeras within the lattice, which leads to BiHTL crosslinking by proximity. **B.** Replicate western blots of purified immature HIV-1 VLPs treated with 500 nM Bivalent Halo Tag Ligand (BiHTL; 1 hour at 37° C). **C.** Model for cell-based chemical crosslinking with BiHTL and EnvCT-containing Halo chimeras: (top) immobile chimeras will remain uncrosslinked, (bottom) IgG-mediated crosslinking of HA epitopes could lead to increased BiHTL crosslinking. **D.** Replicate western blots for HIV-1 infected COS7 cell lysates expressing Halo chimeras. For cell samples, control represents no BiHTL crosslinker treatment and BiHTL condition is treated with 5 μM for 3 hours at 4° C. The +HA-IgG condition is antibody crosslinking of HA-Halo for 1 hour prior to BiHTL, which did not have a significant effect dimerizing chimeras. **E.** Densitometry analysis of VLP western blots demonstrate the relative percentage of dimerized HA-Halo-gt-(EnvCT/ΔCT). **F.** Densitometry of the cellular amount of dimerized HA-Halo-gt-(EnvCT/ΔCT). Overall, under these experimental conditions and with repeated measures, ~99% of VLPs containing Halo probes are largely immobile and residing alone in lattice association sites, distant from one another on the VLPs. These results also suggest that trapping of the Env-CT does not require prior oligomerization. For all western blots, HA-Halo-gt-EnvCT/ΔCT was detected with anti-HA (Clone 3F10), Gag was detected with anti-p24 (183-H12-5C) mAb, and α-tubulin detected with anti-α-Tubulin (B-5-1-2) mAb. All error bars represent SDs. All *P* values > 0.05 are labeled as not significant (ns) using a two-tailed t-test.

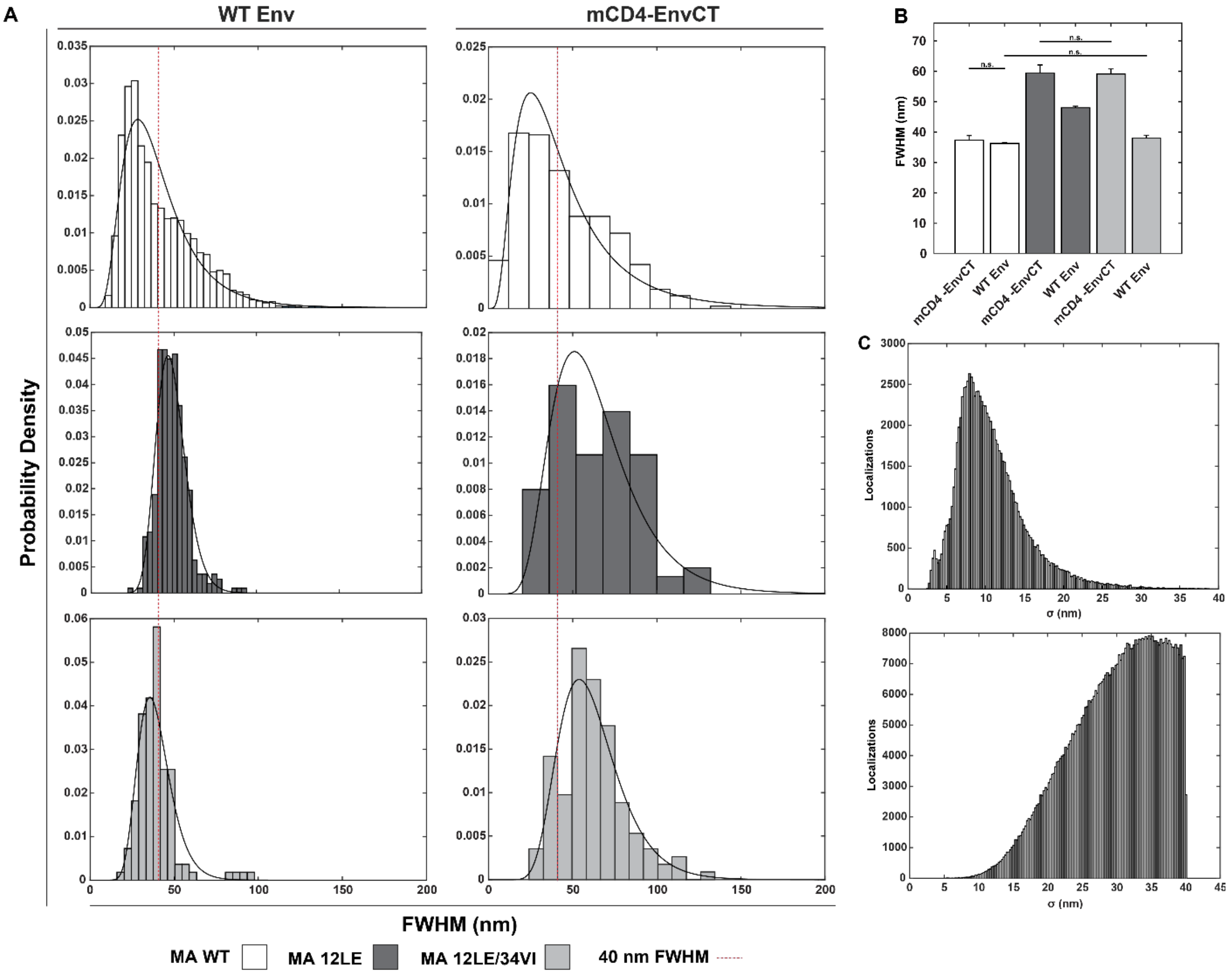

Fig. S5. Cumulative displacements of single molecule trajectories in proximity to HIV-1 assembly sites. A. X- and Y- full-width at half-maximum (FWHM) estimates of individual single molecule trajectory positions at single virus assembly sites. Distributions of the FWHM were fit to a lognormal distribution for both WT Env (left column) and chimeras (right column) proximal to WT (top row), 12LE (middle row) and 12LE/34VI (bottom row) MA lattices. **B.** Non-parametric t-tests were performed across all FWHM distributions. No statistical significance was observed for the displacement of single molecule trajectories for either trimers or chimeric monomers proximal to WT MA, and trimers proximal to 12LE/34VI MA, suggesting that those single molecules trapped at virus assembly sites all exhibit similar confinement (sub-40 nm FWHM distribution). An arbitrary reference of 40 nm FWHM is indicated using a red vertical dash line to highlight distribution shifts for the genotypes listed. **C.** Aggregate single molecule localization precision (σ) for entire study. The top panel is the uncertainties for the QD-625 probe (mean σ=11±4 nm, *n*=95,599) and bottom panel for the CA-SkylanS probe (mean σ=30±7 nm, *n*=580,452) after filtering all localizations with uncertainties above 40 nm.

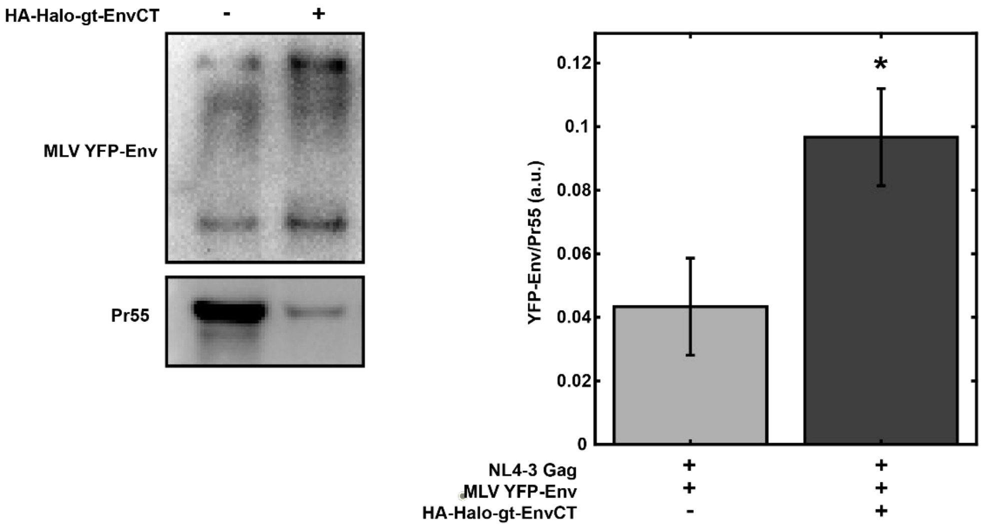

Fig. S6. MLV Env does not compete with monomeric Env-CT chimeras for HIV-1 incorporation. YFP-MLV-Env was co-transfected with Env deleted NL4-3 and HA-Halo-gt-EnvCT into HEK293T cells to examine the strength of competitive inhibition. MLV Env-YFP was detected using an anti-GFP antibody, and degraded YFP-Env is observed as a species that aligns with the molecular weight of YFP. MLV Env incorporation was enhanced by expression of the HA-Halo-gt-EnvCT chimera, rather than being inhibited (*P*=0.019; n=3). With a lack of direct competition, this data suggests that pseudotyping with MLV Env is a passive incorporation mechanism and is distinct from the lattice association sites of HIV-1 Env-CT.

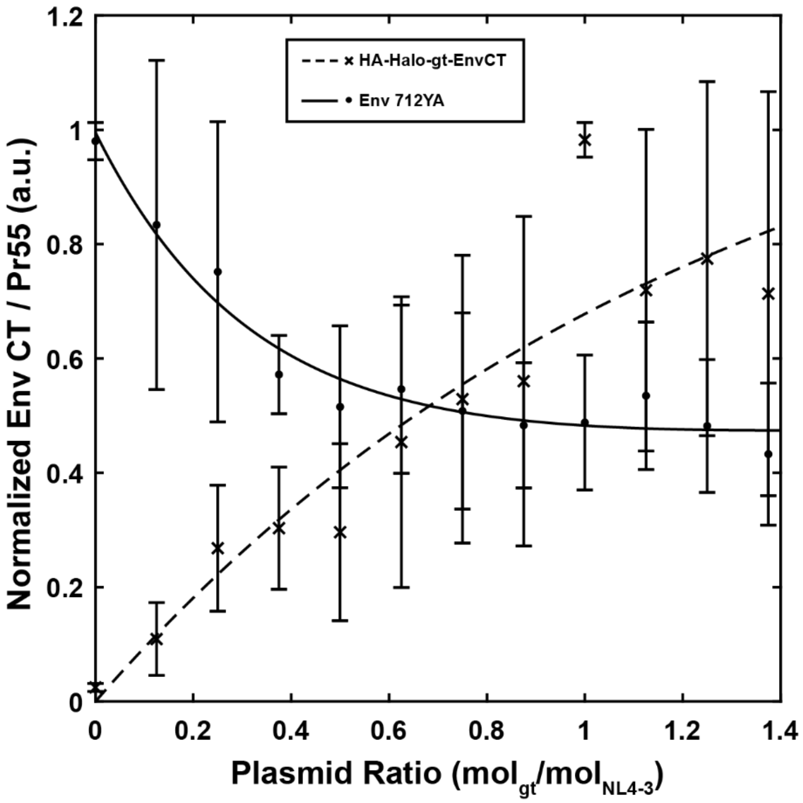

Fig. S7. HA-Halo-gt-EnvCT incorporation is saturable in HEK293T-derived pseudovirus. Increasing amounts of HA-Halo-gt-EnvCT (gt) plasmid were co-transfected into HEK293T cells with pSVNL4-3-*pol*(-)-Env-712YA (NL4-3; Pr55 = Gag). The data points at increasing concentrations were fit to a double exponential recovery curve (HA-Halo-gt-EnvCT curve evaluated at infinity: $Y_{Infinity}$ = 1.24 mol$_{gt}$/mol$_{NL4-3}$, root mean square error = 0.11). The saturation curve of HA-Halo-gt-EnvCt evaluated at the IC_{50} of WT Env (0.6 mol$_{gt}$/mol$_{NL4-3}$) is approximately 0.5 mol$_{gt}$/mol$_{NL4-3}$. The near intersection of these curves at the 712YA Env IC_{50} indicates a similar MA lattice preference for trimers and monomers. The incomplete loss of WT Env during competition may be attributed to shed micro-vesicle contamination or another, trimer specific, incorporation mechanism that cannot be accessed or accommodated by a monomeric Env-CT. Error bars represent SD.
